# CryoPromptSeg: prompt-guided segmentation with integrated denoising for cryo-EM particle picking

**DOI:** 10.1093/bioinformatics/btag327

**Published:** 2026-05-22

**Authors:** Bin Yang, Yujie You, Liang Jin, HongYang Yu, Le Zhang

**Affiliations:** College of Computer Science, Sichuan University, Chengdu, 610065, China; School of Computer Science and Engineering, Sichuan University of Science and Engineering, Yibin, 644000, China; Bioland Laboratory (Former Guangzhou Regenerative Medicine and Health-Guangdong Laboratory), Guangzhou, 510005, China; Bioland Laboratory (Former Guangzhou Regenerative Medicine and Health-Guangdong Laboratory), Guangzhou, 510005, China; SIP-UCLA Institute for Technology Advancement, Suzhou, 215123, China; College of Computer Science, Sichuan University, Chengdu, 610065, China

## Abstract

**Motivation:**

Cryo-electron microscopy (Cryo-EM) single particle analysis (SPA) is a key technique for revealing the structure of biomacromolecules by three-dimensional reconstruction. Achieving high-resolution reconstruction relies on the acquisition of a large number of authentic particles; however, manual particle picking is inefficient and inadequate for the demands of reconstruction, making automated particle picking a major research focus. Although the foundational segmentation model Segment Anything Model (SAM) has recently advanced automated particle picking, its segmentation advantages have not been fully realized in cryo-EM applications. Moreover, cryo-EM images often have significant noise. Conventional denoising decreases noise but frequently overlooks high-level semantic information, leading to oversmoothed particle regions and reduced particle distinguishability.

**Results:**

To address these challenges, we propose CryoPromptSeg, which employs prompt-guided SAM for particle picking while integrating a semantically enhanced image denoiser. Specifically, by performing domain adaptation fine-tuning of SAM and incorporating prompts generated by the proposed automatic prompt generator, it achieves precise segmentation of cryo-EM particles. In addition, it employs a parallel multi-task framework to jointly train the denoiser and the prompt generator, incorporating particle semantic information from the prompt generator into the denoiser to suppress noise while preserving highly distinguishable particle structures. To lower the barrier to practical application, we developed a user-friendly online prediction platform for particle picking. Experimental results demonstrate that CryoPromptSeg outperforms existing mainstream methods in both particle picking accuracy and image denoising quality, thus providing a novel solution for the automation of particle picking.

**Availability:**

The code and platform are available at: https://github.com/347251369/CryoPromptSeg.

## 1 Introduction

Cryo-electron microscopy (Cryo-EM) single particle analysis (SPA) enables the reconstruction of three-dimensional structures of biological macromolecules from two-dimensional projection images collected under cryogenic conditions (Cheng *et al.* 2015). As a result, this technique has been widely applied to the structural elucidation and functional analysis of viruses, proteins, and other complex biological macromolecules. As a breakthrough in structural biology, Cryo-EM single particle analysis enables near-atomic resolution analysis of macromolecular complexes often intractable using conventional techniques (Milne *et al.* 2013). To achieve high-resolution 3D reconstruction, scientists typically need to identify and extract more than 100 000 particle images from cryo-EM micrographs (Wagner *et al.* 2019). However, manual particle picking is not only time-consuming but also heavily influenced by human subjectivity (Dhakal *et al.* 2023).

To address the low efficiency and high subjectivity associated with manual particle picking, extensive research has focused on developing semi-automated or fully automated cryo-EM particle picking methods. Classical approaches often rely on image processing techniques such as template matching (Scheres 2012, Punjani *et al.* 2017) and edge detection (Adiga *et al.* 2005, Woolford *et al.* 2007), but they are highly susceptible to artifacts such as ice contamination, carbon edges, particle overlap, and structural deformation. These factors frequently result in a high false positive rate due to the erroneous identification of non-particle regions as particles. In recent years, with the rapid advancement of deep learning techniques (Jiang *et al.* 2024, Zhang *et al.* 2024, You *et al.* 2025) such as convolutional neural networks (CNNs) and Transformers, neural network-based cryo-EM particle picking models have demonstrated significant improvements in both accuracy and robustness (Wang *et al.* 2016, Tegunov and Cramer 2019, Nguyen *et al.* 2021, Dhakal *et al.* 2025). Among them, CrYOLO (Wagner *et al.* 2019) and Topaz (Bepler *et al.* 2019) have been widely adopted for cryo-EM particle picking. Despite their superior performance over classical methods, these models still exhibit notable limitations: CrYOLO often fails to detect real particles, resulting in an insufficient number of valid particles, while Topaz tends to misidentify false particles, leading to a high false positive rate.

Segment Anything Model (SAM) (Kirillov *et al.* 2023), with its powerful segmentation capability, has provided a new paradigm for the innovation of particle picking techniques (Gyawali *et al.* 2024, He *et al.* 2024). Based on SAM, CryoSegNet (Gyawali *et al.* 2024) innovatively integrates the attention-gated U-Net architecture (Ronneberger *et al.* 2015) into SAM, surpassing the CrYOLO and Topaz models in several benchmark tests. However, current studies have two main limitations: One is when handling a large number of protein particles in cryo-EM images, dense manual prompts are neither practical nor efficient. Therefore, CryoSegNet only uses SAM’s automatic mask generator (Kirillov *et al.* 2023) without adopting its prompt-based interactive segmentation capability, failing to fully leverage the guiding role of prompt information in the segmentation process. The other is that SAM is trained on natural images and excels at segmenting objects with clear structures and relatively large scales. However, since particles in cryo-EM images are typically small and exhibit blurry boundaries, the image encoder of SAM lacks sufficient representation capacity for such tiny targets, making it difficult to directly apply SAM to segmentation in cryo-EM images (Gyawali *et al.* 2024). Thus, we propose our first scientific question: how can a prompt generation model be developed to automatically generate high-quality prompts that guide SAM in image segmentation, while incorporating domain adaptation fine-tuning to effectively increase the performance of cryo-EM particle picking?

In addition, cryo-EM images are short on low contrast and low signal-to-noise ratio (SNR), which decreases the particle distinguishability, resulting in a major challenge to the accuracy of particle picking models (Chung *et al.* 2022). Thus, current studies (Dhakal *et al.* 2024, Gyawali *et al.* 2024) commonly adopt image denoising as a preprocessing step to mitigate the interference of noise on particle picking. However, the denoising methods employed in these studies essentially rely on the pixel-level information of cryo-EM images while neglecting the extraction and utilization of high-level semantic information. As a result, they tend to oversmooth particle regions during noise suppression, leading to the loss of structural information and diminished particle discernibility. Based on these issues, we propose our second scientific question: how can semantic information be effectively incorporated into cryo-EM image denoising to remove background noise while preserving the detailed structural information of particles, thereby increasing image quality?

In recent years, although substantial advances have been achieved in denoising and particle picking algorithms for cryo-EM, and many methods have been released as open-source code or toolkits (Bepler *et al.* 2019, 2020, Wagner *et al.* 2019, George *et al.* 2021), the practical adoption of these methods remains limited mainly due to their reliance on complicated command-line operations and environment configurations. This challenge makes the methods accessible primarily to expert users and raises the barrier for wider application in scientific research. Therefore, we propose our third scientific question: how can we develop a user-friendly platform that integrates image denoising function, particle picking function, and interactive visualization capabilities to increase the convenience of cryo-EM image processing workflows?

To answer the above scientific questions, we propose CryoPromptSeg model and make the following contributions. Firstly, we not only develop an automatic prompt generator (APG) based on an encoder coupled with a feature pyramid network (FPN) (Lin *et al.* 2017a) to provide high-quality point and mask prompts for SAM, but also introduce a multi-cognitive visual adapter (Mona) (Yin *et al.* 2024) into the SAM encoder to increase its representation capability for fine-scale particles by domain adaptation fine-tuning. Secondly, we integrate an image denoiser and the APG with semantic segmentation property into a parallel multi-task learning framework, enabling feature interaction through a selective feature integrator (SFI). Under joint training, the APG provides semantic guidance to the denoiser, while the denoiser reduces background noise interference on the APG. Finally, we develop a user-friendly platform that integrates image denoising, particle picking, and interactive visualization capabilities to process cryo-EM images efficiently and conveniently. We carried out a comprehensive evaluation of our CryoPromptSeg model on the CryoPPP cryo-EM image dataset (Dhakal *et al.* 2023). We demonstrate that our proposed method outperforms the existing mainstream methods in both particle picking accuracy and image denoising quality.

## 2 Methods

**Figure 1:**
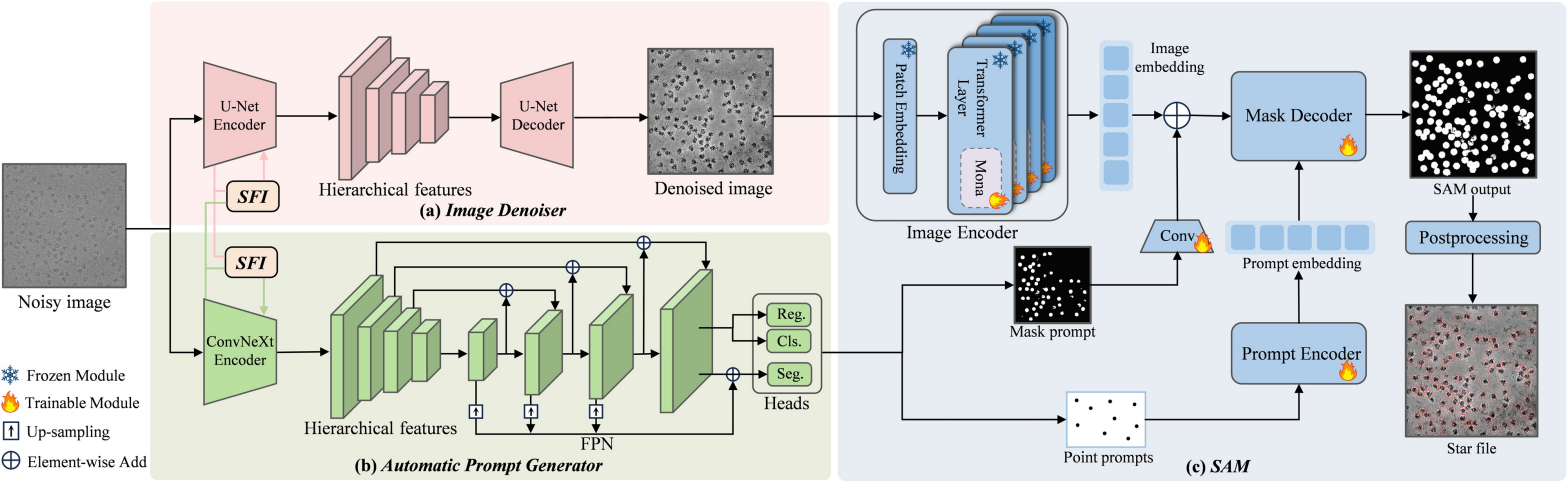
The overall architecture of CryoPromptSeg, which consists of an image denoiser, an automatic prompt generator, a selective feature integrator, and a SAM model. (a) image denoiser, (b) automatic prompt generator, (c) SAM.

Here, we propose CryoPromptSeg, which integrates two tasks: denoising and particle picking. The overall architecture of CryoPromptSeg is described in Fig. 1, which consists of four main modules. (i) The image denoiser is built upon a U-Net structure, aiming to restore high-quality images. (ii) The automatic prompt generator (APG) is composed of a ConvNeXt (Liu *et al.* 2022) encoder, a feature pyramid network (FPN), and multiple prediction heads, which are developed to generate point and mask prompts for SAM. (iii) The selective feature integrator (SFI) enables targeted feature fusion between corresponding layers of the two encoders. (iv) The Segment Anything Model (SAM), equipped with multi-cognitive visual adapter (Mona), employs the generated prompts to make segmentation masks from the denoised image.

### 2.1 Multi-task framework for denoising and prompt generation

**Figure 2:**
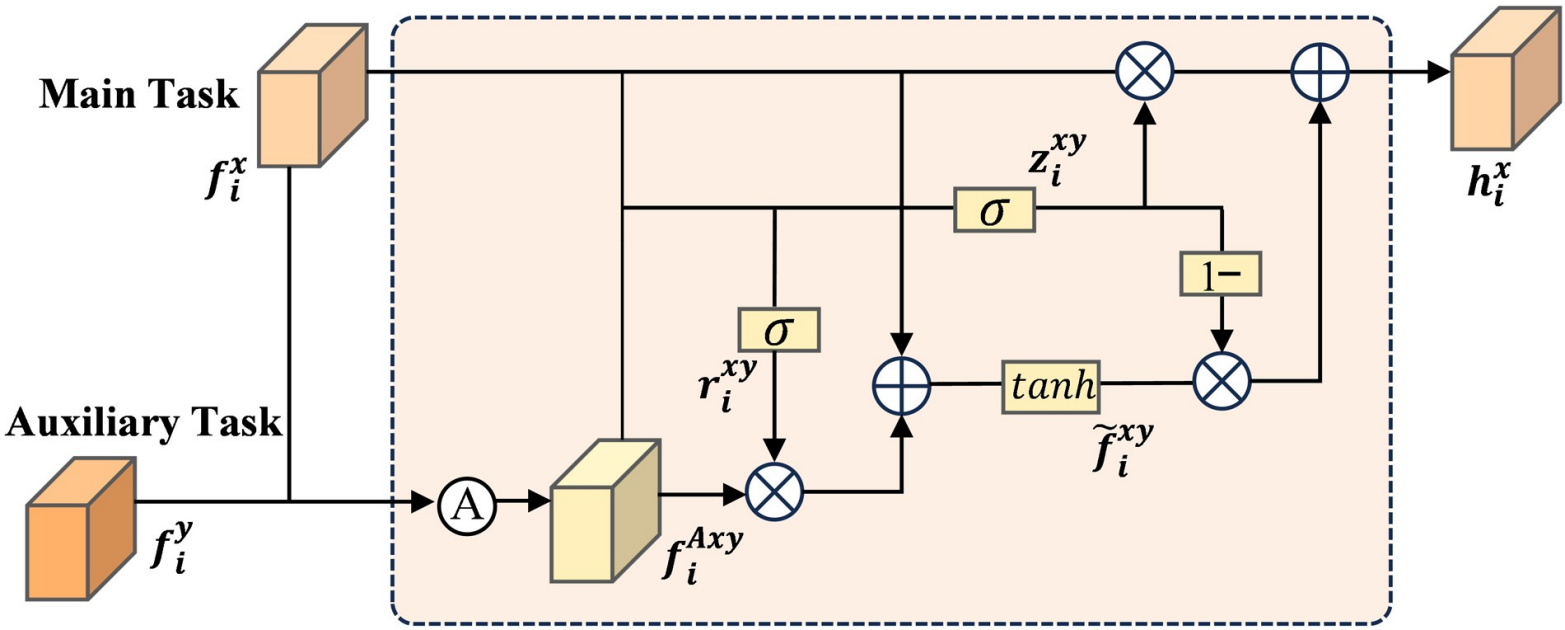
The architecture of the selective feature integrator (SFI). “A” denotes the cross-attention mechanism, σ denotes the sigmoid function, tanh denotes the tanh activation function, ⊗ and ⊕ indicate element-wise multiplication and addition, respectively.

As illustrated in Fig. 1a and b, we propose a multi-task framework comprising two modules: the image denoiser and the APG. These two modules operate in parallel and take the same noisy image as input. The selective feature integrator (SFI) based on gating mechanism is introduced between their respective encoders. Through the SFI, the APG can acquire clearer structural information from the denoiser, whereas the denoiser can obtain richer semantic priors from the APG. This enables a deep interaction and sharing of structural and semantic information between two tasks. The architecture of SFI is shown in Fig. 2. Specifically, we fuse the main task feature $f_{i}^{x}$ with the auxiliary task feature $f_{i}^{y}$ via cross-attention to obtain the fused feature $f_{i}^{\mathrm{Axy}}$. A leaky gate $r_{i}^{xy}$ is then employed to control the incorporation ratio of $f_{i}^{\mathrm{Axy}}$, producing an intermediate representation ${\tilde{f}}_{i}^{xy}$. Finally, a memory gate $z_{i}^{xy}$ balances the main task feature $f_{i}^{x}$ and ${\tilde{f}}_{i}^{xy}$ to generate the final output $h_{i}^{x}$. Detailed computations are provided in Supplementary Note S1. The SFI enables the main task x to selectively fuse beneficial features from the auxiliary task y at the  i-th layer of encoder.

**Figure 3:**
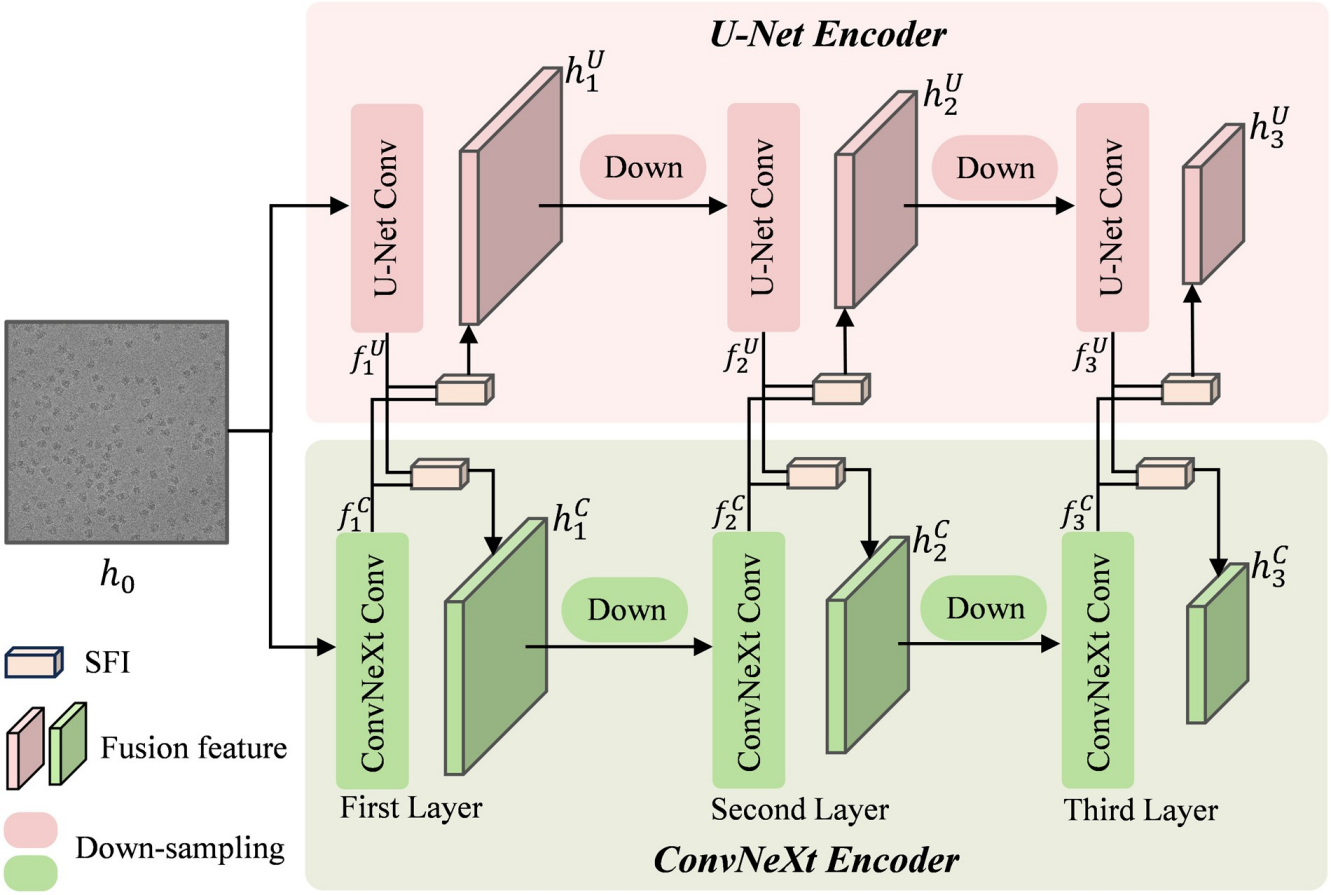
Layer-wise feature interaction and fusion between the encoders.

Given that the image denoiser needs semantic information from the APG, while the APG also relies on structural information from the image denoiser, the information flow between the two modules is bidirectional. To this end, we insert paired SFI modules between the corresponding layers of the U-Net encoder and the ConvNeXt encoder, thereby enabling each task to extract feature representation beneficial to itself from the other, as illustrated in Fig. 3.

Taking the feature flow in the U-Net encoder as an example, the image denoising task serves as the main task, while prompt generation is treated as the auxiliary task. The first convolutional block of the U-Net encoder extracts structural feature $f_{1}^{U}$ from the input image $h_{0}$. These structural feature $f_{1}^{U}$ is then fed along with the semantic feature $f_{1}^{C}$, which is output by the first layer of the ConvNeXt encoder, into the SFI for fusion. The fusion feature $h_{1}^{U}$ is then down-sampled and passed to the next layer. This process, including convolution, fusion via SFI, and down-sampling, is repeated at each layer. Thus, the feature extraction and interaction process at the i-th layer of the U-Net encoder can be described as:


(1) $$h_{i}^{U}=\mathrm{SFI}({\mathrm{Conv}}_{i}^{U}(h_{i-1}^{U}),f_{i}^{C}),$$

where $h_{i}^{U}$ represents the fusion feature at the i-th layer. When i=1,$\;h_{0}^{U}$ refers to the input image $h_{0}$. The term $f_{i}^{C}$ denotes the semantic feature generated by the i-th layer of the ConvNeXt encoder and ${\mathrm{Conv}}_{i}^{U}$ refers to the convolutional block at the i-th layer of the U-Net encoder. Through the SFI module, the denoiser can selectively introduce semantic information from the APG branch. This mechanism helps preserve critical features in regions of interest while effectively suppressing background noise.

Similarly, the feature extraction and interaction process at the i-th layer of the ConvNeXt encoder can be formulated as:


(2) $$h_{i}^{C}=\mathrm{SFI}({\mathrm{Conv}}_{i}^{C}(h_{i-1}^{C}),f_{i}^{U}),$$

where $f_{i}^{U}$ denotes the structural feature extracted by the i-th layer of the U-Net encoder. Through this design, the APG branch can obtain clean structural information from the denoiser, which prevents noise in the input image from interfering with the extraction and interpretation of semantic information.

#### 2.1.1 Image denoiser

The image denoiser aims to remove noise from cryo-EM images and restore clear details. As shown in Fig. 1a, the image denoiser employs a multi-layer encoder-decoder structure of the U-Net network. The noisy image is firstly processed layer by layer through the U-Net encoder, which extracts clean structural feature and interacts with the SFI module at each level. After all encoder layers are processed, the fusion feature from each layer (i.e. $h_{1}^{U}\cdots h_{i}^{U}$, as shown in Fig. 3) are aggregated to form a set of hierarchical feature maps. After that, the decoder, which is symmetric to the encoder, integrates features at different scales through gradual up-sampling and skip connections, ultimately generating a denoised image.

Since noise-free images are difficult to obtain in cryo-EM, supervised denoising methods cannot be directly applied in this setting (Buchholz *et al.* 2019, Bepler *et al.* 2020, Palovcak *et al.* 2020). Therefore, we adopt an unsupervised denoising strategy based on the Noise2Noise algorithm (Lehtinen *et al.* 2018) to train the denoiser $f_{\theta }$. Specifically, the acquired movie frame sequences are divided into even and odd frame sets, which are independently processed and summed following the standard cryo-EM image processing pipeline. This procedure yields a pair of noisy images $\left(x_{\mathrm{even}},x_{\mathrm{odd}}\right)$ that share the same underlying signal but contain independent noise, where $x_{\mathrm{even}}$ and $x_{\mathrm{odd}}$ are obtained by processing and summing the even and odd frame sets, respectively. And then, the model is guided to learn the statistical property of the underlying true signal by minimizing the mean squared error between the denoised output $f_{\theta }(x_{\mathrm{even}})$ and the reference image $x_{\mathrm{odd}}$. The corresponding loss function $L_{\mathrm{denoise}}$ is defined as follows:


(3) $$L_{\mathrm{denoise}}=E_{(x_{\mathrm{even}},x_{\mathrm{odd}})∼\chi }[{\left\|f_{\theta }(x_{\mathrm{even}})-x_{\mathrm{odd}}\right\|}^{2}],$$

where $E_{(x_{\mathrm{even}},x_{\mathrm{odd}})∼\chi }$ denotes the expectation computed over the training set χ, i.e. the average loss.

#### 2.1.2 Automatic prompt generator

Since protein particles of cryo-EM images are typically small in volume and densely distributed, it is easy to encompass adjacent particles when using box as prompt for SAM and in turn compromise segmentation accuracy. On the contrary, the point prompt is more concise and accurate, effectively indicating the particle center. Therefore, we employ center point as spatial prompt. Based on this, we firstly generate a set of uniformly distributed grid coordinates with a fixed stride, according to the size of the input noisy image, to serve as the coordinates of the reference points. And then, the APG will not only predict coordinates corresponding to particle centers by refining and classifying these reference points, but also generate a coarse segmentation mask of the entire image. The predicted center coordinates and the generated segmentation mask are used as point and mask prompts for the SAM model.

As illustrated in Fig. 1b, the APG first employs an encoder (ConvNeXt) to extract semantic feature from the input noisy image. At each layer, the SFI fuses the semantic feature coming from ConvNeXt with the denoised structural feature coming from the image denoiser to yield the noise-suppressed fusion feature $h_{i}^{C}$. And then, it is passed to the next layer. Subsequently, the fusion features ${(h}_{1}^{C}\cdots h_{i}^{C})$ coming from all layers are sent to the FPN. To exploit the multi-level feature information in the feature pyramid, we map the coordinates of each reference point to the corresponding coordinate space of each feature map.

Let the i-th feature map in the FPN be denoted as $F^{\left(i\right)}\in R^{C_{i}\times H_{i}\times W_{i}}$, where i∈[1,L], L is the total number of pyramid levels, $C_{i}$ denotes the number of channels, and the spatial resolution is $H_{i}\times W_{i}$. Given an input image of size H×W, the mapped coordinates $\left(x_{p}^{i},y_{p}^{i}\right)$ of a reference point $\left(x_{p},y_{p}\right)$ on the i-th feature map are computed as follows, where p represents the index of the reference point.


(4) $$(x_{p}^{i},y_{p}^{i})=(x_{p}\cdot \frac{W_{i}}{W},y_{p}\cdot \frac{H_{i}}{H}),$$

On the feature map $F^{\left(i\right)}$, we obtain the feature vector $f^{i}(x_{p}^{i},y_{p}^{i})$ of the reference point by bilinear interpolation (Shui *et al.* 2024). Finally, we form a multi-scale representation $f(x_{p},y_{p})$ of the reference point by concatenating the feature vectors from all levels.


(5) $$f(x_{p},y_{p})=\mathrm{Concat}(f^{1}(x_{p}^{1},y_{p}^{1}),f^{2}(x_{p}^{2},y_{p}^{2}),\ldots ,f^{L}(x_{p}^{L},y_{p}^{L})),$$

The multi-scale representation of each reference point is fed into a regression MLP head and a classification MLP head to predict a set of candidate center point coordinates along with their corresponding confidence scores. To generate a coarse segmentation mask, the feature maps from all FPN levels are up-sampled to a unified spatial resolution and fused via element-wise addition. After that, the aggregated feature map is fed into a segmentation MLP head for mask prediction.

Since the fixed stride is smaller than the size of a particle, multiple predictive points may fall within the same particle. Therefore, the model needs to select the most appropriate one as the particle center. We denote the set of ground-truth points as $P={\left\{p_{i}\right\}}_{i=1}^{N}$, and the set of predicted points as $\hat{P}={\left\{{\hat{p}}_{j}\right\}}_{j=1}^{M}$, where N<M. To establish the optimal correspondence between predicted points and ground-truth points, we adopt the one-to-one matching strategy based on lower distance and higher confidence score proposed by Song *et al.* (2021), using the cost matrix as the basis for pairing. Let $D\in R^{N\times M}$ be the cost matrix for one-to-one matching. The calculation formula for the i-th row and j-th column of the cost matrix $D_{ij}$ is as follows:


(6) $$D_{ij}=\gamma {\left\|p_{i}-{\hat{p}}_{j}\right\|}_{2}-{\hat{c}}_{j},$$

where $D_{ij}$ represents the cost of pairing the predicted point ${\hat{p}}_{j}$ with the ground truth point $p_{i}$, γ is the equilibrium parameter, ${\left\|\cdot \right\|}_{2}$ denotes the $L_{2}$ norm (i.e. Euclidean distance), and ${\hat{c}}_{j}$ represents the confidence score of ${\hat{p}}_{j}$. We use the Hungarian algorithm (Ma *et al.* 2024) to find the minimum cost matching, ensuring that each ground truth point is uniquely associated with the predicted point that is both closest in spatial distance and has the greatest confidence score.

After the matching process, the prompt generator is optimized by minimizing the discrepancies between the selected predicted points and their corresponding ground-truth points in terms of positional coordinates and categorical classification, as well as the errors in segmentation mask prediction. Specifically, the regression branch $L_{\mathrm{reg}}$ employs the Smooth L1 loss (Girshick 2015) (Equation 7); the classification branch $L_{\mathrm{cls}}$ employs a weighted binary cross-entropy loss (Equation 8) and the segmentation branch $L_{\mathrm{seg}}$ adopts the focal loss (Lin *et al.* 2017b) (Equation 9). The final loss $L_{\mathrm{APG}}$ is defined as a weighted combination of these three components (Equation 10).


(7) $$L_{\mathrm{reg}}=\frac{1}{N}\sum_{i=1}^{N}\mathrm{Smooth}\;L1({\hat{p}}_{i},p_{i}),$$

(8) $$L_{\mathrm{cls}}=-\frac{1}{M}\{\sum_{i=1}^{N}\log{\hat{c}}_{i}+\alpha \sum_{i=N+1}^{M}\log(1-{\hat{c}}_{i})\},$$

(9) $$L_{\mathrm{seg}}=\mathrm{Focal}\;\mathrm{Loss}(\hat{m},m),$$

(10) $$L_{\mathrm{APG}}=\lambda_{1}L_{\mathrm{reg}}+\lambda_{2}L_{\mathrm{cls}}+\lambda_{3}L_{\mathrm{seg}},$$

Specifically, the Smooth L1 loss is defined as:


$$\mathrm{Smooth}\;L1(\hat{p},\;p)=\{\begin{array}{l} 0.5{\left(\hat{p}-p\right)}^{2},\;if|\hat{p}-p|<1\;\\ |\hat{p}-p|-0.5,\;\mathrm{otherwise} \end{array}$$

where $\left|\hat{p}-p\right|$ denotes the absolute error. ${\hat{c}}_{i}$ represents the confidence score of ${\hat{p}}_{i}$, and α is a weighting factor. $\hat{m}$ and m denote the predicted and ground-truth mask, respectively. λ represents the weighting coefficients for each loss branch.

#### 2.1.3 Multi-task joint training

To avoid unstable feature representation and task interference caused by direct joint training, and to improve overall training effectiveness, we employ a progressive training strategy. Specifically, we first train the image denoiser independently according to Equation (3), enabling it to generate clean image with clear structures and rich details. Subsequently, we employ the denoised image as input to independently train the APG by Equation (10), allowing it to extract semantic information and generate high-quality point and mask prompts. During the separate training scenarios, the SFI module is disabled, and the output of each encoder layer is directly down-sampled and passed to the next layer. Finally, based on the pretrained sub-networks, we activate the SFI module to integrate structural and semantic features, and optimize the entire multi-task framework.

### 2.2 SAM fine-tuning

Figure 1c shows that the denoised cryo-EM image (from Fig. 1a) is fed into the image encoder to extract image embedding. The embedding is then added element-wise to the mask prompt (from Fig. 1b) and fed into the mask decoder. In parallel, point prompts are processed by the prompt encoder to obtain prompt embedding that is also input to the decoder. The mask decoder then generates particle segmentation result, which is further postprocessed to generate a standard .star file containing the coordinates of protein particles. The complete postprocessing procedure is detailed in Supplementary Note S2.

**Figure 4:**
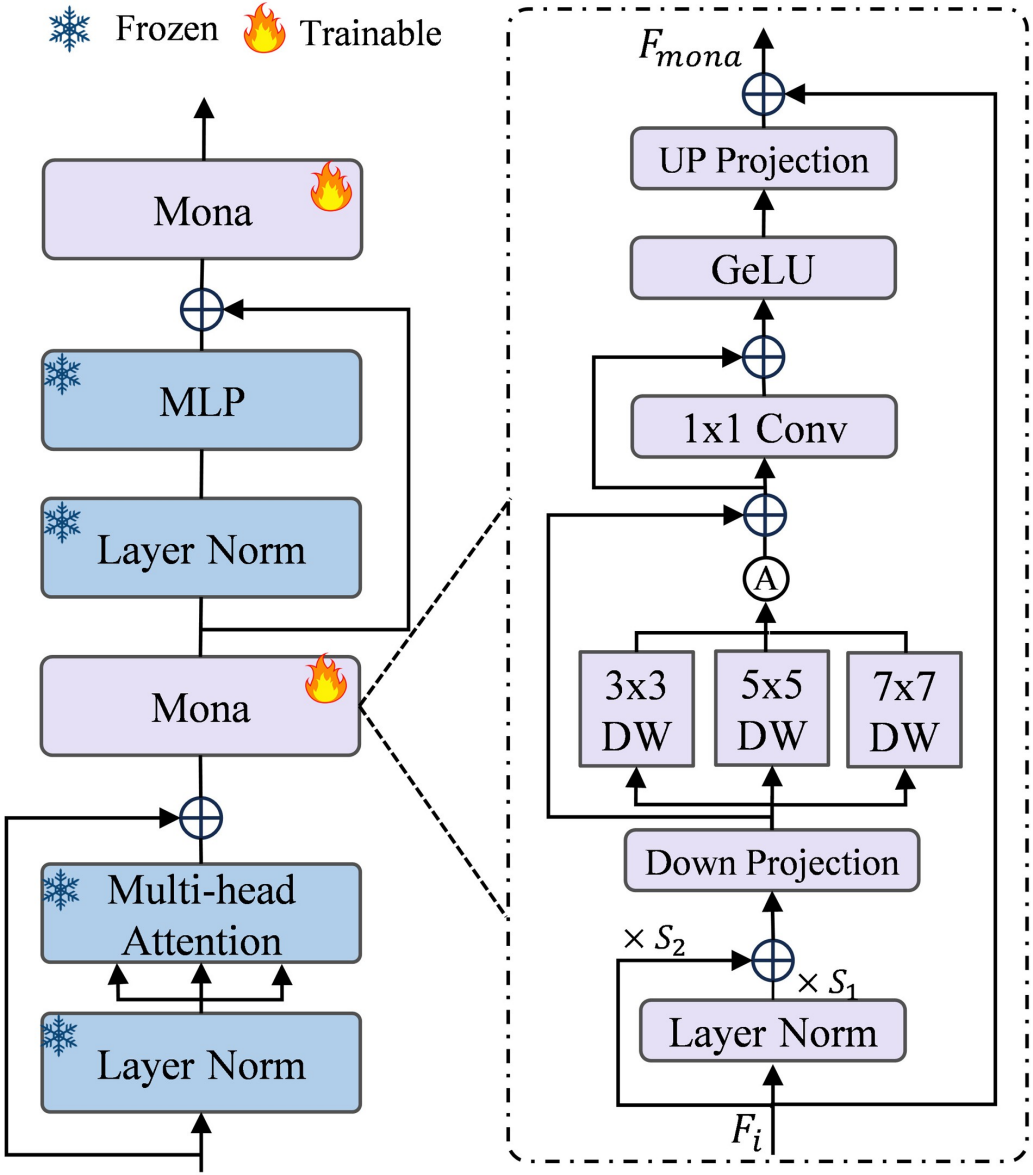
Transformer block in the SAM image encoder augmented with Mona.

To effectively fine-tune the SAM encoder, we introduce trainable multi-cognitive visual adapter (Mona) between the transformer layers, as illustrated in Fig. 4. Mona (Yin *et al.* 2024) enhances the segmentation of small-scale targets in SAM by incorporating multi-scale convolutions that extract local details under different receptive fields in parallel, thereby increasing the representation of tiny particle features for cryo-EM image. Specifically, given the intermediate feature $F_{i}$ from a transformer layer, we apply normalization and weighted combination followed by a down-projection to obtain a low-dimensional representation. Three groups of depthwise separable convolutions are then employed to extract local features. Finally, the features are up-projected and combined with a residual connection to produce the output $F_{\mathrm{mona}}$. Details are provided in Supplementary Note S3.

During fine-tuning, the weights of the SAM encoder remain frozen, while only the parameters of the adapter module, the prompt encoder, and the mask decoder are updated. The segmentation mask predicted by the mask decoder is optimized by the loss $L_{\mathrm{SAM}}$ defined using Equation (11).


(11) $$L_{\mathrm{SAM}}=\lambda_{f}L_{\mathrm{focal}}+L_{\mathrm{dice}}$$

where $L_{\mathrm{focal}}$ denotes the focal loss, $L_{\mathrm{dice}}$ is the Dice loss (Milletari *et al.* 2016), and $\lambda_{f}$ is a coefficient that balances the two loss terms.

### 2.3 Datasets and experimental setup

We collected micrograph samples from 22 protein categories in the CryoPPP dataset, identified by their EMPIAR IDs (Iudin *et al.* 2016). Fifteen categories were used for model training and validation, with 80% of each category assigned to the training set and 20% to the validation set. Detailed sample statistics are in Table S1. The remaining 7 categories served as an independent test dataset to compare CryoPromptSeg with external methods, as detailed in Table S2. Based on the above datasets, we conducted systematic experiments on CryoPromptSeg. The data processing and model parameters are provided in Supplementary Note S4.

## 3 Results

### 3.1 Evaluation metrics

Given the general absence of clean ground truth images in cryo-EM, we evaluate the denoising performance of our model using the SNR metric proposed in Topaz-Denoise (Bepler *et al.* 2020). The evaluation procedure is detailed in Supplementary Note S5. For the particle picking task, we compare the particles picked by CryoPromptSeg with the ground truth coordinates of the expert-labeled particles. Specifically, performance is evaluated using precision, recall, and F1-score. A predicted particle is considered a true positive (TP) if it uniquely matches a ground-truth particle with an IoU>0.5; otherwise, it is a false positive (FP). The Dice score (Bertels *et al.* 2019) is used to assess segmentation performance by measuring the overlap between the SAM-predicted and ground-truth masks.

### 3.2 Particle picking

To answer our first scientific question, we compared the proposed CryoPromptSeg with four representative methods: the widely used CrYOLO and Topaz, as well as two recently developed models, CryoTransformer and CryoSegNet. The comparison was carried out on two levels: one is machine learning metrics such as precision, recall, and so on, the other is the resolution of protein density maps reconstructed from the selected particles. To guarantee a fair comparison, all methods are trained on the same dataset. The detailed information on the parameters used for these four methods can be found in Supplementary Note S6.

#### 3.2.1 The performance of particle picking in terms of machine learning metrics

Table 1 summarizes the quantitative comparison. The first column lists EMPIAR IDs, followed by performance on Precision, Recall, F1-score, and Dice score. Table 1 shows that CryoPromptSeg outperforms CrYOLO, Topaz, CryoTransformer, and CryoSegNet in average Recall (0.794), F1-score (0.738), and Dice score (0.708). Furthermore, we performed a *t*-test (Xiao *et al.* 2024, Li *et al.* 2025) between CryoPromptSeg and other models, with the results shown in Figs. S1–S3. In nearly all datasets, the differences in Recall, F1-score, and Dice score between CryoPromptSeg and the other methods are statistically significant (*P*-value ≤0.05). Specifically, the greatest Recall indicates that the method effectively segments more true particles with a less miss rate; the greatest F1-score reflects the model’s ability to maintain the greatest Recall rate while achieving a good balance with precision; and the greatest Dice score demonstrates its segmentation masks can more accurately anchor the protein locations than others.

**Table 1:** Particle picking results across different test datasets (The best results are highlighted in bold; mean ± standard deviation).

EMPIAR ID	Metrics	CrYOLO	Topaz	CryoTransformer	CryoSegNet	CryoPromptSeg
10028	Precision	0.733 ± 0.037	0.573 ± 0.044	0.635 ± 0.044	0.772 ± 0.054	**0.780 ± 0.031**
	Recall	0.922 ± 0.012	0.896 ± 0.022	**0.923 ± 0.011**	0.874 ± 0.049	0.863 ± 0.038
	F1-Score	0.816 ± 0.024	0.698 ± 0.033	0.752 ± 0.031	0.819 ± 0.047	**0.819 ± 0.013**
	Dice Score	0.807 ± 0.032	0.683 ± 0.044	0.743 ± 0.035	0.811 ± 0.048	**0.820 ± 0.040**
10081	Precision	0.772 ± 0.028	0.645 ± 0.060	0.657 ± 0.053	**0.826 ± 0.060**	0.798 ± 0.028
	Recall	0.804 ± 0.024	0.807 ± 0.046	0.875 ± 0.030	0.835 ± 0.071	**0.903 ± 0.023**
	F1-Score	0.788 ± 0.021	0.714 ± 0.036	0.749 ± 0.030	0.830 ± 0.061	**0.847 ± 0.017**
	Dice Score	0.756 ± 0.055	0.664 ± 0.082	0.689 ± 0.106	0.794 ± 0.083	**0.798 ± 0.089**
10345	Precision	0.428 ± 0.114	0.370 ± 0.078	0.351 ± 0.100	**0.643 ± 0.089**	0.636 ± 0.057
	Recall	0.158 ± 0.078	0.400 ± 0.095	**0.874 ± 0.025**	0.732 ± 0.078	0.856 ± 0.024
	F1-Score	0.223 ± 0.090	0.375 ± 0.067	0.491 ± 0.107	0.682 ± 0.074	**0.727 ± 0.039**
	Dice Score	0.195 ± 0.091	0.314 ± 0.112	0.407 ± 0.071	0.635 ± 0.128	**0.675 ± 0.112**
10532	Precision	0.636 ± 0.064	0.598 ± 0.071	0.566 ± 0.092	**0.704 ± 0.111**	0.691 ± 0.051
	Recall	0.720 ± 0.029	**0.870 ± 0.038**	0.652 ± 0.049	0.552 ± 0.131	0.774 ± 0.034
	F1-Score	0.673 ± 0.038	0.706 ± 0.054	0.599 ± 0.042	0.613 ± 0.114	**0.729 ± 0.035**
	Dice Score	0.647 ± 0.057	0.691 ± 0.061	0.572 ± 0.06	0.567 ± 0.124	**0.709 ± 0.049**
11056	Precision	0.650 ± 0.032	0.653 ± 0.038	0.649 ± 0.025	**0.705 ± 0.041**	0.699 ± 0.032
	Recall	0.736 ± 0.017	**0.868 ± 0.030**	0.589 ± 0.037	0.551 ± 0.058	0.808 ± 0.026
	F1-Score	0.690 ± 0.017	0.744 ± 0.018	0.616 ± 0.018	0.617 ± 0.045	**0.748 ± 0.014**
	Dice Score	0.682 ± 0.016	0.738 ± 0.018	0.610 ± 0.019	0.610 ± 0.053	**0.741 ± 0.018**
10093	Precision	0.545 ± 0.032	0.335 ± 0.067	0.388 ± 0.031	**0.611 ± 0.093**	0.503 ± 0.027
	Recall	0.646 ± 0.032	0.099 ± 0.042	**0.729 ± 0.016**	0.442 ± 0.107	0.663 ± 0.038
	F1-Score	**0.591 ± 0.023**	0.150 ± 0.054	0.506 ± 0.027	0.511 ± 0.100	0.571 ± 0.020
	Dice Score	**0.575 ± 0.031**	0.155 ± 0.057	0.495 ± 0.026	0.505 ± 0.127	0.531 ± 0.053
10017	Precision	0.757 ± 0.043	0.763 ± 0.045	0.738 ± 0.044	**0.833 ± 0.077**	0.773 ± 0.042
	Recall	0.505 ± 0.041	**0.883 ± 0.086**	0.481 ± 0.052	0.563 ± 0.044	0.690 ± 0.062
	F1-Score	0.603 ± 0.023	**0.815 ± 0.048**	0.580 ± 0.033	0.670 ± 0.045	0.727 ± 0.032
	Dice Score	0.586 ± 0.042	**0.804 ± 0.072**	0.557 ± 0.046	0.653 ± 0.068	0.684 ± 0.073
Average	Precision	0.646	0.562	0.569	**0.728**	0.697
	Recall	0.642	0.689	0.732	0.650	**0.794**
	F1-Score	0.626	0.600	0.613	0.677	**0.738**
	Dice Score	0.607	0.578	0.582	0.654	**0.708**

**Figure 5:**
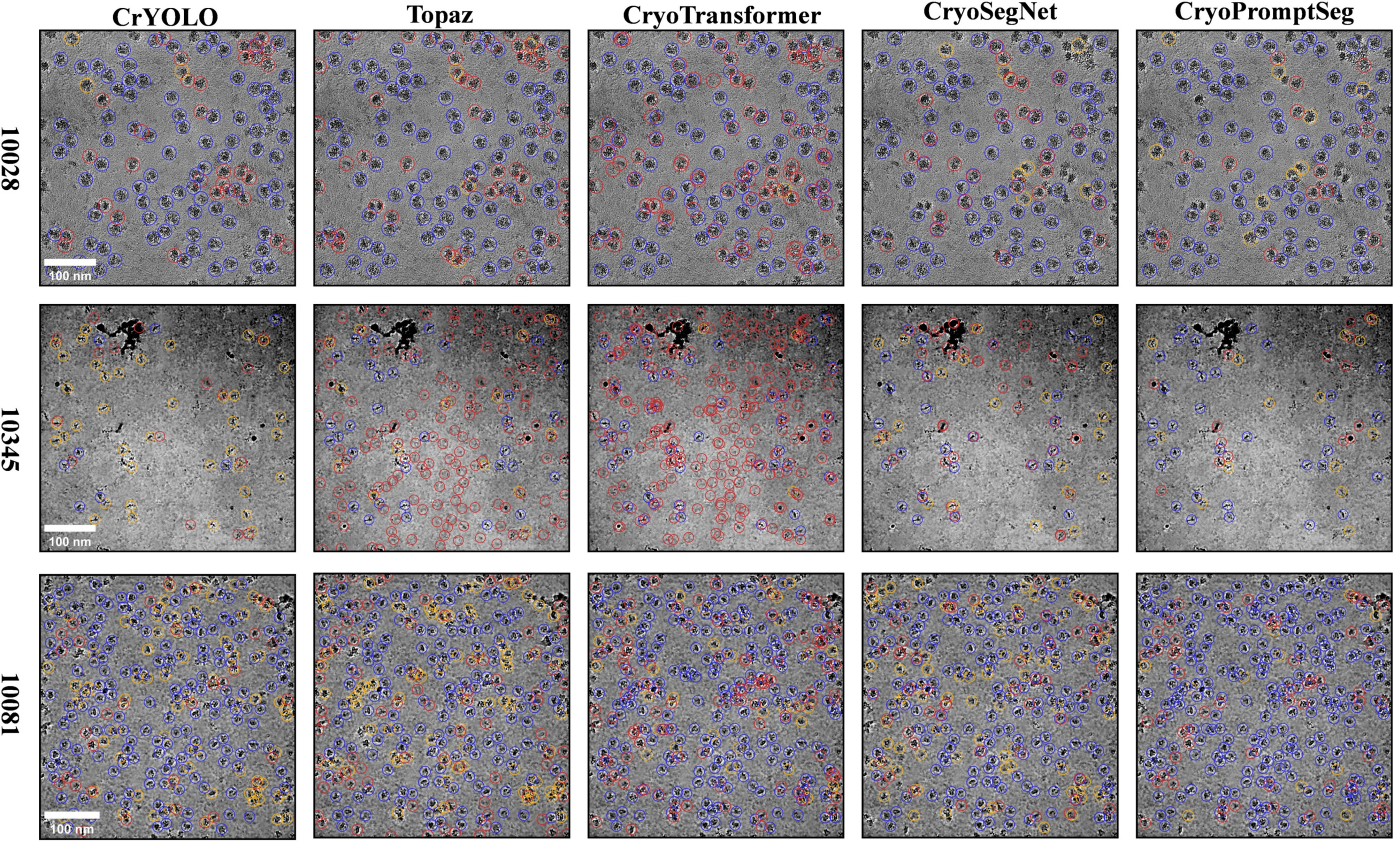
Visualization of particle picking results. Blue circles indicate correctly identified particles (true positives), red circles denote incorrectly identified particles (false positives), and yellow circles represent missed true particles (false negatives).

In addition, Fig. 5 visualizes the particle picking results for each method on micrographs of three proteins. Figure 5 shows that CrYOLO selects fewer particles, leading to a greater number of false negatives and noticeable missed detections. Topaz and CryoTransformer show fewer missed particles but generate more false positives. CryoSegNet often generates repeated predictions at the same particle locations. In contrast, CryoPromptSeg effectively identifies most true particles while maintaining low false positive and false negative rates, which demonstrates that CryoPromptSeg has more stable performance and better overall effectiveness for particle picking.

#### 3.2.2 The performance of particle picking in terms of reconstruction resolution

Machine learning metrics evaluate model performance but don’t directly reflect the quality of the protein density map reconstructed from the selected particles. Resolution is a key indicator of reconstruction quality, since higher resolution implies better particle selection and reconstruction. Thus, we compared the resolutions of 3D density maps reconstructed by CrYOLO, Topaz, CryoTransformer, CryoSegNet, and CryoPromptSeg on the same test datasets. The 3D reconstruction and resolution estimation were performed using CryoSPARC (Punjani *et al.* 2017). For each method and each protein, the experiment was repeated three times, and the average resolution was used for comparison. The reconstruction procedure is detailed in Supplementary Note S7, and the specific experimental results are listed in Table S3.

Table 2 lists the average resolution (in Å) of density map obtained from 3D reconstruction across different datasets for each method. As shown, CryoPromptSeg achieved the highest resolution on six out of the seven datasets, indicating that it is more effective and has better generalization capability to select high-quality particles that enable high-resolution reconstructions across most proteins.

**Table 2:** Comparison of the average reconstruction resolutions across different test datasets [resolution (Å); the best results are highlighted in bold].

Model	10028	10081	10345	10532	11056	10093	10017
CrYOLO	3.96	3.93	6.00	3.60	8.87	4.13	**4.30**
Topaz	4.09	4.05	6.03	3.64	8.11	5.90	4.60
CryoTransformer	4.00	4.26	5.71	3.78	7.41	4.57	4.76
CryoSegNet	4.09	4.00	6.21	3.86	8.05	5.41	5.33
**CryoPromptSeg**	**3.86**	**3.62**	**5.11**	**3.51**	**7.08**	**3.72**	4.46

### 3.3 Micrograph denoising

To answer our second scientific question, we conducted a comparative study against two methods to evaluate the effectiveness of CryoPromptSeg in the denoising task. One is the classical Low-pass filtering method, and the other is the Topaz-Denoise method (Bepler *et al.* 2020), which is based on the Noise2Noise framework. In CryoPromptSeg, denoising results are produced by the denoiser embedded within its multi-task framework.

The results are shown in Table 3, where the first column lists the EMPIAR IDs, and the remaining columns report average SNR values for different denoising methods (including raw images), along with paired *t*-test results comparing each method with CryoPromptSeg. Detailed SNR distributions and *t*-test results for each dataset are in Fig. S4.

**Table 3:** Quantitative comparison of denoising results [SNR (dB); higher value indicates lower noise level; the best results are highlighted in bold].

EMPIAR ID	Raw		Low-pass		Topaz		Ours
	SNR	*P*-value	SNR	*P*-value	SNR	*P*-value	SNR
10017	−14.46	$7.6\times 10^{-8}$	−4.9	$1.1\times 10^{-5}$	−1.18	$2.7\times 10^{-3}$	**1.85**
10028	−9.44	$1.5\times 10^{-9}$	−2.4	$2.3\times 10^{-7}$	1.68	$2.5\times 10^{-4}$	**4.2**
10081	−20.33	$1.2\times 10^{-10}$	−1.65	$1.3\times 10^{-6}$	3.62	$1.7\times 10^{-6}$	**6.81**
10093	−21.71	$2.6\times 10^{-8}$	−6.39	$1.3\times 10^{-3}$	−6.04	$2.3\times 10^{-3}$	**−3.48**
10345	−16.83	$6.6\times 10^{-10}$	0.89	$7.5\times 10^{-8}$	5.66	$2.2\times 10^{-4}$	**9.03**
10532	−16.52	$9.8\times 10^{-9}$	−4.88	$3.3\times 10^{-5}$	−2.25	$2.9\times 10^{-3}$	**0.82**
11056	−33.89	$4.1\times 10^{-12}$	−6.48	$1.9\times 10^{-4}$	−6.71	$1.5\times 10^{-5}$	**−2.3**

Table 3 shows that CryoPromptSeg increases SNR by approximately 21 dB on average over the raw images. Compared to the Low-pass and Topaz-Denoise, CryoPromptSeg yields SNR gains of approximately 6.1 dB and 3.2 dB, respectively. Since Table 3 and Fig. S4 show that *P*-values are generally less than 0.05, the statistical results indicate that the differences of SNR between CryoPromptSeg and the other two methods are statistically significant. Among all methods, CryoPromptSeg consistently has the greatest SNR for all datasets, which are highlighted in bold, and strongly demonstrates the superior denoising performance of CryoPromptSeg across diverse datasets.

**Figure 6:**
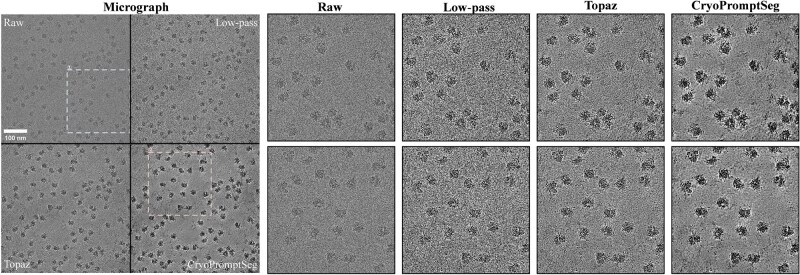
Qualitative comparison of denoising results.

To more intuitively illustrate the denoising performance for each method, Fig. 6 provides qualitative comparisons across the full micrograph and two magnified regions (the magnified regions are marked by dashed boxes in the full micrograph). Figure 6 shows that the low-pass filtering method retains substantial background noise after denoising and exhibits limited noise suppression, but the deep learning-based methods, CryoPromptSeg and Topaz, demonstrate better performance in both noise reduction and background smoothing. Notably, CryoPromptSeg not only effectively removes background noise, but also preserves key structural details of particles. As a result, particle contours appear clearer and their shapes more prominent for CryoPromptSeg, whereas Topaz exhibits residual noise in some particle regions and tends to over-smooth details during denoising, which results in less prominent particle morphology. Thus, these observations indicate that the incorporation of semantic information enables the denoiser to more effectively distinguish particle signals from background interference, thereby achieving more targeted noise removal.

### 3.4 The cryo-EM particle picking prediction platform

**Figure 7:**
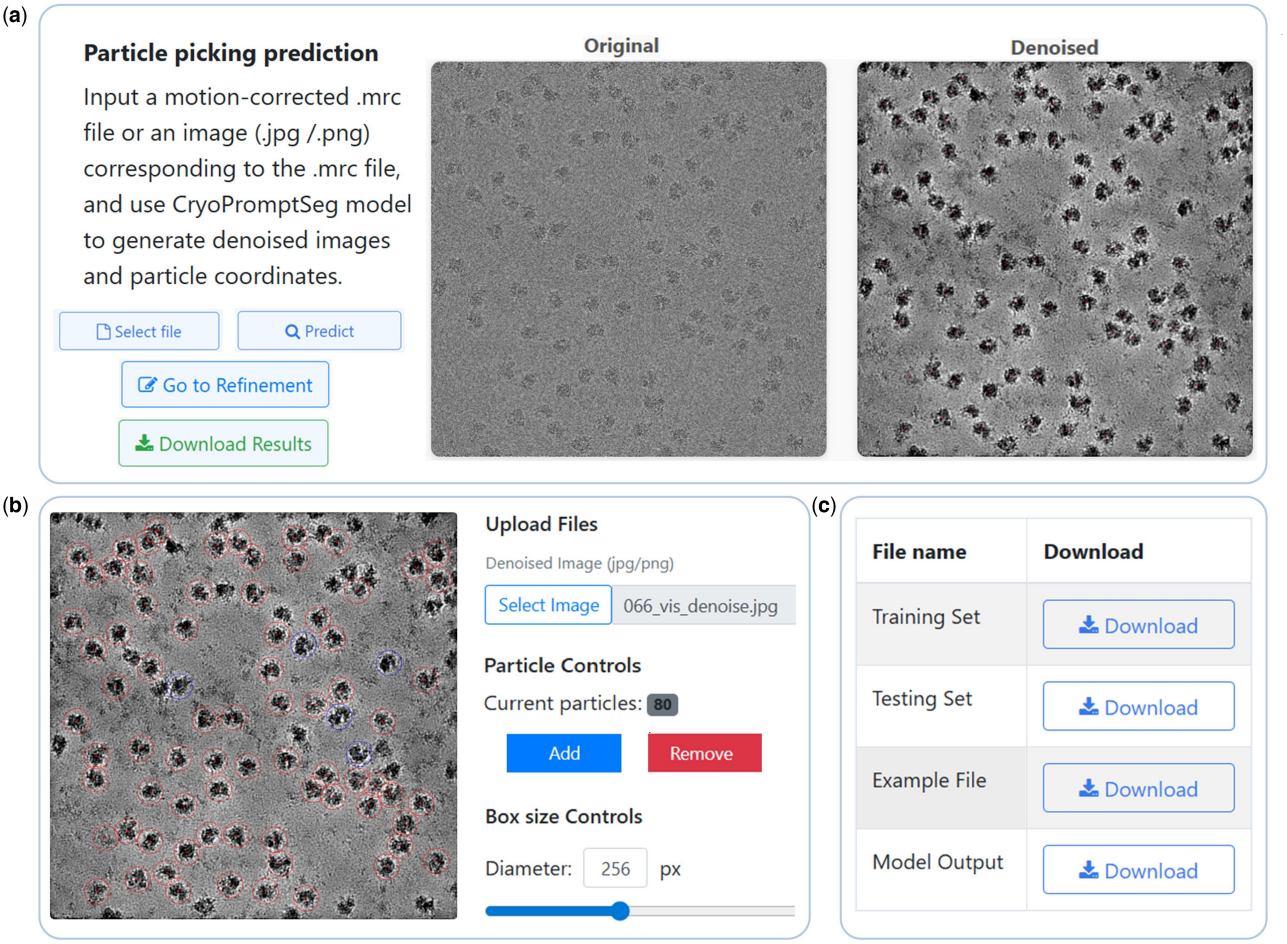
The Cryo-EM particle picking prediction platform. (a) The particle coordinate prediction module, (b) The interactive refinement module, (c) The data download module.

To answer our third scientific question, we developed an online predictive platform based on CryoPromptSeg with three key functions: particle coordinate prediction, interactive refinement, and data download. Figure 7a shows the particle coordinate prediction module, where users upload files and generate denoised images and particle coordinates in “.star” format. Figure 7b illustrates the interactive refinement module, allowing users to add or remove particles and adjust bounding box diameter. Red boxes show model predictions, and blue boxes indicate user-added particles. Figure 7c presents the data download module, where users can click “Download” to obtain the data.

### 3.5 Ablation study and other experiments

We conducted a series of ablation studies to analyze the impact of different prompt types (point-only, mask-only, and combined prompts), various multi-task framework designs, and fine-tuning techniques of the SAM model on particle picking performance, the detailed results of which are provided in Supplementary Note S8.

We grouped the test datasets by particle size, SNR, and particle density to assess our method’s performance across different complexity scenarios, the detailed results of which are provided in Supplementary Note S9. In addition, we compared the training and inference time of different methods under the same configuration settings, the detailed results of which are listed in Supplementary Note S10.

## 4 Conclusion

In this paper, we propose a novel particle picking model, CryoPromptSeg, which integrates an automatic prompt generator and a Mona fine-tuned SAM. Guided by high-quality point and mask prompts, the fine-tuned SAM can accurately select particles from cryo-EM micrographs. To mitigate the over-smoothing of particle regions during denoising, CryoPromptSeg develops an image denoiser parallel to the prompt generator. By incorporating the semantic information from the prompt generator through a selective feature integrator, it effectively suppresses noise while increasing particle discriminability, thereby aiding the model in identifying and picking particles. However, the model’s performance is limited by the scale and diversity of the training data. Future research will focus on expanding the high-quality and diverse cryo-EM annotated dataset to improve the model’s generalization capability. Additionally, SAM lacks classification ability, which may lead to the misidentification of non-particle structures as particles. Future work will explore integrating a classifier into SAM to increase particle picking accuracy.

## Supplementary Material

btag327_Supplementary_Data

## Data Availability

The code and data are available at: https://github.com/347251369/CryoPromptSeg.
