# Supplementary material for: CryoPromptSeg: prompt-guided segmentation with integrated denoising for cryo-EM particle picking
Source: Bioinformatics. 2026 May 22;42(6):btag327. doi: 10.1093/bioinformatics/btag327 (PMC13241001; doi:10.1093/bioinformatics/btag327)
Supplement: btag327_Supplementary_Data [file btag327_supplementary_data.pdf]

SUPPLEMENTAL INFORMATION FOR

CryoPromptSeg: Prompt-guided Segmentation with Integrated Denoising for Cryo-EM Particle Picking

content

1. Supplementary Tables 2
    1.1 The dataset used for training, validation, and testing 2
    1.2 The results of reconstruction resolution 4
    1.3 The ablation study results of the multi-task framework 5
    1.4 The ablation study results of the fine-tuning 6
    1.5 The ablation study results of the prompt type 7
    1.6 Testing dataset characteristics and complexity levels 8
    1.7 Training and inference time 9
2. Supplementary Notes 10
    2.1 Supplementary Note S1: Computation process of SFI 10
    2.2 Supplementary Note S2: Postprocessing of the output of SAM 11
    2.3 Supplementary Note S3: Computation process of Mona 11
    2.4 Supplementary Note S4: Data processing and model parameters 12
    2.5 Supplementary Note S5: SNR calculation process 13
    2.6 Supplementary Note S6: Parameters of comparison methods 13
    2.7 Supplementary Note S7: 3D reconstruction 14
    2.8 Supplementary Note S8: Ablation study 16
        2.8.1 Impact of different task frameworks 16
        2.8.2 Impact of different fine-tuning techniques 17
        2.8.3 Impact of different prompt types 18
    2.9 Supplementary Note S9: Performance under different complexity scenarios 19
    2.10 Supplementary Note S10: Training and inference time comparison 20
3. Supplementary Figures 21
    3.1 Distribution of Recall in different datasets 21
    3.2 Distribution of F1-score in different datasets 22
    3.3 Distribution of Dice score in different datasets 23
    3.4 Distribution of SNR in different datasets 24
    3.5 Examples of particle and background regions 25
    3.6 Selected 2D classes of protein particles 26
    3.7 Comparison of 3D density maps 27
    3.8 Additional reconstruction results 28
    3.9 Comparison of local resolution estimation 30
    3.10 Visualization results of denoising and prompt generation 31
    3.11 Visualization of the three-dimensional feature space 32

1. Supplementary Tables

1.1 The dataset used for training, validation, and testing

Supplementary Table S1: An overview of the dataset used for training and validation of CryoPromptSeg.

EMPIAR ID	Type of Protein	Image Size	Training Images	Validation Images
10005 (Liao et al. 2013)	TRPV1 Transport Protein	(3710, 3710)	23	6
10059 (Gao et al. 2016)	TRPV1 Transport Protein	(3838, 3710)	232	59
10075 (Koning et al. 2016)	Bacteriophage MS2	(4096, 4096)	239	60
10077 (Fischer et al. 2016)	Ribosome (70S)	(4096, 4096)	240	60
10096 (Tan et al. 2017)	Viral Protein	(3838, 3710)	240	60
10289 (Burendei et al. 2020)	Transport Protein	(3710, 3838)	240	60
10406 (Nicholson et al. 2020)	Ribosome (70S)	(3838, 3710)	191	48
10444 (Demura et al. 2020)	Membrane Protein	(5760, 4092)	236	60
10590 (Mashtalir et al. 2020)	TRPV1 Transport Protein	(3710, 3838)	236	60
10737 (Li et al. 2021)	Membrane Protein	(5760, 4092)	233	59
10760 (Kuzuya et al. 2022)	Membrane Protein	(3838, 3710)	240	60
10816 (Oldham et al. 2016)	Transport Protein	(7676, 7420)	240	60
10852 (Cao et al. 2021)	Signaling Protein	(5760, 4092)	274	69
11051 (Newing et al. 2020)	Transcription/DNA/RNA	(3838, 3710)	240	60
11183 (Liu et al. 2023)	Signaling Protein	(5760, 4092)	240	60
	Total		3344	881

Supplementary Table S2: An overview of the independent dataset for testing CryoPromptSeg.

**EMPIAR ID**	**Type of Protein**	**Image Size**	**Number of Images**
10017 (Scheres 2015)	β -galactosidase	(4096, 4096)	84
10028 (Wong et al. 2014)	Ribosome (80S)	(4096, 4096)	300
10081 (Lee and MacKinnon 2017)	Transport Protein	(3710, 3838)	300
10093 (Jin et al. 2017)	Membrane Protein	(3838, 3710)	295
10345 (Campbell et al. 2020)	Signaling Protein	(3838, 3710)	295
10532 (Tan and Rubinstein 2020)	Viral Protein	(4096, 4096)	300
11056 (Asami et al. 2022)	Transport Protein	(5760, 4092)	305
	Total		1,879

1.2 The results of reconstruction resolution

Supplementary Table S3: Comparison of reconstruction resolutions across different test datasets. Bold indicates the best resolution among the three independent runs, while the bold and underlined text indicates the best average resolution across all methods.

EMPIAR ID	Number of Images	Method	Without Select 2D						With Select 2D					
			Number of Particles	Resolution for 3 Trials (Å) (FSC=0.143)			Average Resolution (Å)	P-value	Number of Particles	Resolution for 3 Trials (Å) (FSC=0.143)			Average Resolution (Å)	P-value
				1	2	3				1	2	3		
10028	300	CrYOLO	31,420	**4.01**	4.02	4.01	4.01	1.9×10^{-3}	25,765	**3.93**	3.98	3.96	3.96	2.8×10^{-2}
		Topaz	47,505	**4.11**	4.11	4.13	4.12	1.0×10^{-3}	25,355	4.13	**4.07**	4.08	4.09	1.0×10^{-2}
		CryoTransformer	37,405	4.03	**4.01**	4.01	4.02	1.5×10^{-2}	27,657	4.01	**4.0**	4.0	4.00	7.0×10^{-3}
		CryoSegNet	25,452	4.09	**4.13**	4.11	4.11	2.6×10^{-3}	22,595	4.10	**4.08**	4.08	4.09	4.1×10^{-3}
		CryoPromptSeg	33,860	**3.93**	3.94	3.94	**3.94**	---	30,542	**3.85**	3.85	3.88	**3.86**	---
10081	300	CrYOLO	45,503	**4.20**	4.23	4.23	4.22	1.7×10^{-1}	32,591	3.93	**3.89**	3.98	3.93	6.8×10^{-3}
		Topaz	58,800	**4.44**	5.01	4.56	4.67	7.2×10^{-2}	40,396	4.08	4.05	**4.02**	4.05	1.6×10^{-3}
		CryoTransformer	72,181	4.92	4.90	**4.86**	4.89	1.6×10^{-2}	53,657	**4.16**	4.34	4.28	4.26	6.8×10^{-3}
		CryoSegNet	46,467	4.38	**4.31**	4.49	4.39	4.1×10^{-2}	32,969	4.0	**3.95**	4.05	4.00	5.7×10^{-3}
		CryoPromptSeg	45,801	**3.89**	4.05	4.20	**4.05**	---	38,519	**3.62**	3.62	3.62	**3.62**	---
10345	295	CrYOLO	16,256	**6.91**	7.19	7.28	7.13	2.1×10^{-1}	11,316	6.01	6.0	**5.98**	6.00	5.0×10^{-4}
		Topaz	43,141	**7.97**	8.29	8.35	8.20	3.9×10^{-2}	22,110	6.05	6.06	**5.97**	6.03	1.2×10^{-3}
		CryoTransformer	80,292	**8.05**	8.38	8.39	8.27	3.5×10^{-2}	44,377	**5.32**	5.95	5.85	5.71	8.1×10^{-2}
		CryoSegNet	22,591	8.16	8.16	**7.70**	8.01	1.9×10^{-2}	13,260	6.01	**5.94**	6.69	6.21	4.4×10^{-2}
		CryoPromptSeg	23,515	6.96	**6.24**	6.29	**6.50**	---	16,954	**5.08**	5.13	5.11	**5.11**	---
10532	300	CrYOLO	46,577	3.71	3.68	**3.64**	3.68	3.1×10^{-1}	28,011	3.60	3.61	**3.59**	3.60	3.9×10^{-2}
		Topaz	59,066	**3.97**	3.97	4.04	3.99	4.6×10^{-3}	35,815	**3.61**	3.66	3.65	3.64	5.1×10^{-2}
		CryoTransformer	113,666	5.13	**4.63**	5.0	4.92	1.6×10^{-2}	63,690	3.76	**3.75**	3.83	3.78	1.2×10^{-2}
		CryoSegNet	31,336	5.83	**5.68**	6.63	6.05	1.4×10^{-2}	21,986	**3.83**	3.89	3.87	3.86	8.3×10^{-3}
		CryoPromptSeg	37,124	**3.58**	3.65	3.64	**3.62**	---	26,269	3.54	**3.49**	3.51	**3.51**	---
11056	305	CrYOLO	64,072	**9.09**	9.42	9.64	9.38	6.2×10^{-3}	57,715	9.03	8.91	**8.67**	8.87	4.8×10^{-3}
		Topaz	46,503	**8.85**	9.0	9.15	9.00	3.0×10^{-3}	43,166	8.08	**7.84**	8.40	8.11	2.2×10^{-2}
		CryoTransformer	118,415	8.23	**7.52**	7.63	7.79	3.3×10^{-1}	98,735	7.47	**7.27**	7.48	7.41	4.9×10^{-2}
		CryoSegNet	69,827	9.32	**8.45**	8.83	8.87	3.9×10^{-2}	60,563	**7.89**	8.23	8.03	8.05	8.0×10^{-3}
		CryoPromptSeg	70,230	**7.43**	7.54	7.46	**7.48**	---	57,827	**7.04**	7.09	7.11	**7.08**	---
10093	295	CrYOLO	45,514	**6.10**	6.21	6.19	6.17	3.9×10^{-4}	35,437	**4.11**	4.12	4.15	4.13	8.7×10^{-4}
		Topaz	31,681	**6.24**	6.74	6.92	6.63	1.1×10^{-2}	20,887	6.06	**5.81**	5.82	5.90	1.4×10^{-3}
		CryoTransformer	117,371	6.67	**6.53**	6.54	6.58	3.9×10^{-3}	72,710	**4.17**	4.31	5.23	4.57	1.2×10^{-1}
		CryoSegNet	47,234	**7.0**	7.18	7.56	7.25	4.9×10^{-3}	32,421	5.49	5.55	**5.18**	5.41	6.0×10^{-3}
		CryoPromptSeg	47,152	**4.97**	5.15	5.07	**5.06**	---	38,568	3.72	**3.69**	3.75	**3.72**	---
10017	84	CrYOLO	53,912	4.35	4.34	**4.33**	**4.34**	5.0×10^{-3}	39,729	**4.28**	4.32	4.29	**4.30**	2.4×10^{-2}
		Topaz	29,887	4.75	4.70	**4.67**	4.71	2.4×10^{-1}	21,021	4.61	**4.59**	4.60	4.60	2.1×10^{-2}
		CryoTransformer	32,210	5.22	**5.18**	5.28	5.23	4.0×10^{-4}	23,212	4.76	**4.75**	4.78	4.76	1.6×10^{-3}
		CryoSegNet	11,709	5.87	5.76	**5.42**	5.68	2.0×10^{-2}	8,439	5.31	**5.30**	5.37	5.33	1.5×10^{-5}
		CryoPromptSeg	41,909	4.64	**4.62**	4.68	4.65	---	29,501	**4.44**	4.44	4.50	4.46	---

1.3 The ablation study results of the multi-task framework

Supplementary Table S4: Ablation study results of the multi-task framework.

Model	Metrics	10028	10081	10345	10532	11056	10093	10017
W/O MTL	Precision	0.775	0.804	0.438	0.639	0.681	0.404	0.772
	p-value	3.7×10^{-3}	2.3×10^{-2}	1.6×10^{-22}	3.9×10^{-9}	9.9×10^{-5}	2.6×10^{-24}	3.7×10^{-2}
	Recall	0.617	0.791	0.689	0.742	0.672	0.744	0.666
	p-value	2.6×10^{-24}	1.2×10^{-17}	3.7×10^{-18}	1.3×10^{-16}	8.3×10^{-29}	7.9×10^{-7}	3.3×10^{-2}
	F1-Score	0.681	0.796	0.536	0.687	0.675	0.521	0.71
	p-value	5.7×10^{-18}	1.9×10^{-14}	1.1×10^{-25}	8.8×10^{-21}	7.0×10^{-21}	3.2×10^{-9}	6.0×10^{-2}
W/O SFI	Precision	0.674	0.802	0.257	0.627	0.688	0.368	0.765
	p-value	9.8×10^{-25}	6.1×10^{-3}	6.0×10^{-32}	2.5×10^{-14}	1.2×10^{-3}	3.8×10^{-27}	5.0×10^{-3}
	Recall	0.529	0.837	0.504	0.696	0.71	0.518	0.599
	p-value	4.9×10^{-21}	2.7×10^{-13}	4.3×10^{-25}	2.1×10^{-25}	2.1×10^{-26}	5.0×10^{-12}	2.5×10^{-26}
	F1-Score	0.576	0.819	0.33	0.660	0.698	0.426	0.672
	p-value	1.2×10^{-19}	7.9×10^{-10}	7.2×10^{-32}	1.4×10^{-26}	7.4×10^{-19}	4.7×10^{-21}	2.6×10^{-20}
**CryoPromptSeg**	Precision	0.78	0.798	0.636	0.691	0.699	0.503	0.773
	Recall	0.863	0.903	0.856	0.774	0.808	0.663	0.69
	F1-Score	0.819	0.847	0.727	0.729	0.748	0.571	0.727

1.4 The ablation study results of the fine-tuning

Supplementary Table S5: Ablation study of different fine-tuning techniques. (Dice score; best results are highlighted in bold).

**Method**	**Metrics**	**10028**	**10081**	**10345**	**10532**	**11056**	**10093**	**10017**
Pretrained SAM	Dice Score	0.484	0.354	—	0.435	0.533	—	—
	p-value	6.3×10^{-30}	5.9×10^{-42}	—	4.4×10^{-14}	5.2×10^{-28}	—	—
FT SAM	Dice Score	0.663	0.608	0.324	0.542	0.625	0.259	0.481
	p-value	5.4×10^{-26}	2.6×10^{-29}	1.7×10^{-36}	6.8×10^{-12}	2.2×10^{-25}	4.8×10^{-48}	5.1×10^{-24}
Adapter SAM	Dice Score	0.801	0.756	0.581	0.605	0.734	0.444	0.468
	p-value	1.7×10^{-8}	4.9×10^{-10}	3.6×10^{-28}	1.6×10^{-10}	7.6×10^{-2}	5.2×10^{-37}	2.2×10^{-27}
**CryoPromptSeg**	Dice Score	**0.82**	**0.798**	**0.675**	**0.709**	**0.741**	**0.531**	**0.684**

1.5 The ablation study results of the prompt type

Supplementary Table S6: Ablation study of different prompt types. (The best results are highlighted in bold).

Metrics	Prompt	10028	10081	10345	10532	11056	10093	10017
Precision	point-only	0.742±0.038	0.716±0.105	0.579±0.115	0.672±0.085	0.676±0.029	0.466±0.053	**0.798±0.081**
	mask-only	0.707±0.042	0.687±0.1	0.475±0.141|	0.603±0.079	0.603±0.079	0.403±0.036	0.687±0.075
	combination	**0.780±0.031**	**0.798±0.028**	**0.636±0.057**	**0.691±0.051**	**0.699±0.032**	**0.503±0.027**	0.773±0.042
Recall	point-only	0.862±0.02	**0.908±0.019**	0.852±0.09	0.738±0.088	0.805±0.046	**0.679±0.049**	0.654±0.034
	mask-only	0.832±0.031	0.901±0.014	0.834±0.02	0.709±0.023	0.786±0.022	0.593±0.021	0.612±0.023
	combination	**0.863±0.038**	0.903±0.023	**0.856±0.024**	**0.774±0.034**	**0.808±0.026**	0.663±0.038	**0.690±0.062**
F1-Score	point-only	0.809±0.028	0.793±0.083	0.676±0.121	0.700±0.063	0.735±0.022	0.553±0.036	0.722±0.063
	mask-only	0.786±0.03	0.772±0.083	0.610±0.161	**0.731±0.063**	0.711±0.022	0.537±0.029	0.677±0.063
	combination	**0.819±0.013**	**0.847±0.017**	**0.727±0.039**	0.729±0.035	**0.748±0.014**	**0.571±0.020**	**0.727±0.032**
Dice Score	point-only	**0.823±0.027**	0.759±0.072	0.633±0.116	0.682±0.062	0.732±0.024	0.518±0.039	0.642±0.054
	mask-only	0.777±0.027	0.742±0.064	0.609±0.143	0.709±0.049	0.735±0.015	0.511±0.027	0.643±0.057
	combination	0.820±0.040	**0.798±0.089**	**0.675±0.112**	**0.709±0.049**	**0.741±0.018**	**0.531±0.053**	**0.684±0.073**

Supplementary Table S7: Paired T-test results to compare the point-only and mask-only with combined prompts, respectively. (p) denotes the p-value for each metric.

Paired *T*-test	Metrics	10028	10081	10345	10532	11056	10093	10017
point-only – combination	Precision (p)	1.2×10^{-33}	7.6×10^{-21}	1.1×10^{-20}	1.3×10^{-3}	3.0×10^{-34}	5.7×10^{-12}	3.0×10^{-28}
	Recall (p)	7.1×10^{-16}	1.4×10^{-14}	1.3×10^{-13}	9.5×10^{-11}	1.4×10^{-15}	8.6×10^{-19}	6.9×10^{-18}
	F1-Score (p)	4.6×10^{-5}	1.1×10^{-11}	3.3×10^{-21}	1.8×10^{-4}	4.0×10^{-6}	3.8×10^{-4}	4.4×10^{-20}
	Dice Score(p)	8.9×10^{-12}	4.1×10^{-3}	5.1×10^{-20}	2.0×10^{-12}	9.4×10^{-3}	7.7×10^{-21}	9.2×10^{-22}
mask-only – combination	Precision (p)	6.1×10^{-21}	8.6×10^{-24}	1.3×10^{-5}	4.5×10^{-23}	4.2×10^{-44}	4.3×10^{-23}	1.5×10^{-21}
	Recall (p)	4.9×10^{-9}	7.7×10^{-3}	3.6×10^{-16}	4.2×10^{-19}	1.8×10^{-28}	7.3×10^{-27}	4.7×10^{-2}
	F1-Score (p)	3.3×10^{-10}	1.7×10^{-21}	2.7×10^{-2}	1.2×10^{-3}	7.5×10^{-15}	5.7×10^{-15}	3.4×10^{-30}
	Dice Score(p)	2.4×10^{-15}	1.5×10^{-15}	3.3×10^{-3}	4.3×10^{-3}	7.0×10^{-3}	5.8×10^{-10}	4.9×10^{-30}

1.6 Testing dataset characteristics and complexity levels

Supplementary Table S8: Testing dataset characteristics and complexity (including particle size, signal-to-noise ratio, particle density, overall complexity distance, and CryoPromptSeg F1-score).

EMPIAR ID	Diameter	SNR	Density	Distance	F1-Score	Scene
10028	224	-9.44	5	0.0467	0.819	low-complexity
10081	154	-20.33	9	0.7698	0.847	medium-complexity
10345	149	-16.83	4	0.7137	0.727	
10532	174	-16.52	18	0.6792	0.729	
10093	172	-21.71	14	0.9201	0.571	
11056	164	-33.89	18	1.2085	0.748	high-complexity
10017	108	-14.46	36	1.4290	0.727	

1.7 Training and inference time

Supplementary Table S9: Comparison of training and inference time across different methods.

Metric	CrYOLO	Topaz	CryoTransformer	CryoSegNet	CryoPromptSeg
Training Time	20min	27min	23min	17min	28min
Inference Time	0.55s	3.14s	5.21s	8.89s	3.87s

2. Supplementary Notes

2.1 Supplementary Note S1: Computation process of SFI

We use the main task feature f_i^x as the query, and the auxiliary task feature f_i^y as the key and value. By computing cross-attention, we obtain the fused feature f_i^{Axy}, which preliminarily extracts useful information from the auxiliary task.

$$f_i^{Axy} = \mathrm{CrossAttn}\left(f_i^x, f_i^y\right) = \mathrm{softmax}\left(\frac{QK^T}{\sqrt{d_k}}\right)V, \quad (1)$$

where $Q = \mathrm{Conv_q}(f_i^x)$, $K = \mathrm{Conv_k}(f_i^y)$, $V = \mathrm{Conv_v}(f_i^y)$, each Conv denotes a learnable convolutional kernel, and d_k is the dimensionality of the key vectors. Then, we incorporate gating mechanisms to further select and control the integration of f_i^{Axy}.

The features f_i^{Axy} and f_i^x are fed into the leaky gate r_i^{xy} to further select key information from f_i^{Axy}, as shown below:

$$r_i^{xy} = \sigma\left(\mathrm{Conv_r}([f_i^x, f_i^{Axy}])\right), \quad (2)$$

where σ denotes the sigmoid function, and $[\,]$ indicates concatenation along the channel dimension. The leaky gate r_i^{xy} is used to control the incorporation ratio of the feature f_i^{Axy}, thereby guiding the fusion between f_i^{Axy} and f_i^x, as shown below:

$$\tilde{f}_i^{xy} = \tanh\left(\mathrm{Conv_U}(f_i^x) + \mathrm{Conv_W}(r_i^{xy} \odot f_i^{Axy})\right), \quad (3)$$

where $\tanh$ and $\odot$ denote the tanh activation function and element-wise multiplication, respectively. In addition, a memory gate z_i^{xy} is employed to control the relative weighting between f_i^x and $\tilde{f}_i^{xy}$ in the final output h_i^x. The specific fusion process is as follows:

$$z_i^{xy} = \sigma\left(\mathrm{Conv_z}([f_i^x, f_i^{Axy}])\right), \quad (4)$$

$$h_i^x = z_i^{xy} \odot f_i^x + \left(1 - z_i^{xy}\right) \odot \tilde{f}_i^{xy}. \quad (5)$$

2.2 Supplementary Note S2: Postprocessing of the output of SAM

In the postprocessing stage, we retain only the segmentation masks from the SAM model (Kirillov et al. 2023) with a confidence score which is greater than 0.5, and use them as the input, along with the point prompt coordinates generated by the automatic prompt generator. Subsequently, OpenCV is employed to perform contour detection on the segmentation mask, extracting all external contours. Each external contour corresponds to a connected region (i.e., connected component (He et al. 2017)) in the mask, representing a potential particle candidate.

To remove small noise fragments, we further filter the candidate contours based on their areas, retaining only those with an area greater than 0.3 times the mean contour area. Next, all prompt points are examined: if a point falls within a given contour, its coordinates are recorded and the corresponding contour is deleted to avoid double-counting. For the remaining contours, the center of the minimum bounding rectangle is computed and taken as the centroid of the particle. Finally, the set of centroids together with the recorded prompt point coordinates constitutes the final particle coordinate set, which is saved in a .star file for subsequent downstream analysis.

2.3 Supplementary Note S3: Computation process of Mona

Given the intermediate feature F_i from a transformer layer as input, we first perform layer normalization ($|F_i|_{LN}$) and apply a weighted combination by learnable scaling factors s_1 and s_2, which are defined by Eq. 6. The resulting feature is then passed through a down-projection operator to obtain a low-dimensional representation F_{down}. Next, as described in Eq. 7, three groups of depthwise separable convolutions ($\mathrm{DWConv_i}$) are applied to F_{down} in parallel to extract features. The outputs are averaged and added back to F_{down} to obtain F_{dw}. Finally, as shown in Eq. 8, F_{dw} is processed by a 1×1 convolution and a GeLU activation function, followed by an up-projection operator to restore the original dimensionality. The final output F_{mona} is obtained via residual addition between F_i and the up-projected feature.

$$F_{down} = \mathrm{Down}(s_1 \cdot |F_i|_{LN} + s_2 \cdot F_i), \tag{6}$$

$$F_{dw} = F_{down} + \mathrm{Average}\left(\sum_{i=1}^{3} \mathrm{DWConv_i}(F_{down})\right), \tag{7}$$

$$F_{mona} = F_i + \mathrm{Up}\left(\mathrm{GeLU}\left(F_{dw} + \mathrm{Conv_{1x1}}(F_{dw})\right)\right), \tag{8}$$

2.4 Supplementary Note S4: Data processing and model parameters

In our CryoPromptSeg model, the image denoiser employs a five-layer encoder-decoder architecture. The automatic prompt generator employs a ConvNeXt-Small encoder, the number of feature pyramid levels L of which is set to 4. Reference points are uniformly distributed across the image at 8-pixel intervals. The equilibrium term γ in the matching process of Eq. 6 is set to 0.1. In Eq. 8, the modulation factor α for the classification loss is set to 0.2. The loss weights λ_1, λ_2, and λ_3 in Eq. 10 are set to 5e-3, 1.0, and 1.0, respectively. The weight coefficient λ_f in the loss function for SAM fine-tuning (Eq. 11) is fixed at 20.

In the multi-task framework, we use the Adam optimizer by setting an initial learning rate of $1e^{-4}$ and a weight decay of $1e^{-4}$. The batch size is set to 4, and the maximum number of training epochs is 200. Learning rate scheduling follows the CosineAnnealingLR strategy, which gradually decays the learning rate from $1e^{-4}$ to $5e^{-5}$ over the course of training.

During the fine-tuning of SAM, only the base model (SAM-B) is updated due to the memory constraints for GPU. The Adam optimizer is used by setting an initial learning rate of $1e^{-4}$, and training is conducted for a total of 100 epochs. To accelerate convergence, the learning rate is reduced by half at the 50th and 75th epochs.

Given the varying sizes of cryo-EM images across different protein categories, all images are resized to a uniform resolution of 1024 × 1024 pixels. During the training process, data augmentation is carried out by random geometric transformations such as flipping and rotation. For the ground truth segmentation masks required for model training, we followed the approach of Gyawali et al. (Gyawali et al. 2024). Based on the ground truth coordinate files in .csv format, which have particle centers and corresponding diameters, we generated an individual circular mask for each micrograph.

2.5 Supplementary Note S5: SNR calculation process

First, 10 images are randomly selected from each dataset. For each image, 20 pairs of particle and background regions are extracted based on annotated ground-truth particle center labels. The background regions are chosen to be as close as possible to their corresponding particle regions. Example images of selected particle and background regions are shown in the Supplementary Figure S5. Given N particle-background pairs (x_p^i, x_b^i), indexed by i, the mean and variance of each particle and background region are computed. After that, we have u_p^i, v_p^i for particle and u_b^i, v_b^i for background, respectively. The average SNR is then calculated in decibels (dB), as defined by Eq. 9.

$$\mathrm{SNR} = \frac{10}{N}\sum_{i=1}^{N} \log_{10} \frac{(u_p^i - u_b^i)^2}{v_b^i}, \tag{9}$$

2.6 Supplementary Note S6: Parameters of comparison methods

When using CrYOLO (Wagner et al. 2019), we keep particles with a confidence score greater than 0.3 and choose the "PhosaurusNet" architecture due to its effectiveness in detecting small particles. For Topaz (Bepler et al. 2019), particles are extracted using the ResNet16 architecture with a "radius of extracted regions" setting to 12, except for EMPIAR IDs 10093 and 10017, where the radius is set to 15. CryoTransformer (Dhakal et al. 2024) keeps only particles with confidence scores between the 25th and 100th percentiles. CryoSegNet (Gyawali et al. 2024) keeps masks with confidence scores exceeding 88.0.

2.7 Supplementary Note S7: 3D reconstruction

For each dataset, we used CryoPromptSeg to predict particle coordinates and saved the results as .star files that can be imported into CryoSPARC (Punjani et al. 2017) for three-dimensional reconstruction of protein density map. The reconstruction workflow consists of the following steps:

1. Import Micrographs and Particles: We import motion-corrected micrographs with CTF estimation, together with the .star files containing the predicted particle coordinates, into CryoSPARC.

2. Particle Extraction: We extract particles from the micrographs using a specified box size.

3. 2D Classification: We group the extracted particles into different orientation classes based on their projection features. For each class, metrics such as the number of particles and resolution are reported.

4. Select 2D Classes: We identify and discard low-quality or aberrant particles while keeping high-quality and representative ones, thereby providing more accurate and reliable input data for subsequent three-dimensional reconstruction. We illustrate selected 2D classes from several datasets in Supplementary Figure S6.

5. Ab-initio Reconstruction: We generate an initial three-dimensional model directly from the selected particle images without using any prior structural model or starting reference, which serves as the basis for further refinement.

6. Homogeneous Refinement: We refine the initial model into a high-resolution density map by correcting particle defocus and higher-order aberrations, thereby increasing both reconstruction quality and resolution.

During the "Select 2D Classes" step, particles are screened based on class resolution, visual quality, and prior knowledge, making the process inherently subjective. As a result, 3D reconstructions are performed in two modes: with 2D particle selection (Select 2D Classes) and without it. Supplementary Table S3 summarizes the number of particles used for reconstruction, the resolutions from three independent runs, the corresponding average resolution, and the related paired T-test results against CryoPromptSeg for each method under both modes. The results show that CryoPromptSeg achieves the best average resolution across six datasets

(EMPIAR IDs: 10028, 10081, 10345, 10532, 11056, and 10093) regardless of whether 2D selection is applied, and ranks second only on EMPIAR-10017, but it is slightly inferior to CrYOLO. The improvements in resolution are statistically significant ($p \leq 0.05$). In addition, comparison between the two modes indicates that applying the “Select 2D Classes” step in density map reconstruction can further improve the resolution for all methods.

After the reconstruction process, we obtained 3D density maps for multiple proteins. Supplementary Figure S7 presents the 3D reconstruction results based on different particle picking methods: CryoPromptSeg, CrYOLO, Topaz, CryoTransformer, and CryoSegNet. It can be observed that the particles selected by CryoPromptSeg yielded 3D density maps with complete structure and high resolution. Additional results of CryoPromptSeg during the reconstruction process are listed by Supplementary Figure S8-S9, including viewing direction plots and 3D resolution CSFSC curves.

In the subsequent analysis, we performed local resolution estimation for the reconstructed 3D density map. The high local resolution indicates that the structural details in the corresponding region are well defined and can be reliably interpreted, whereas the low resolution suggests that the information in that region is limited. For visualization, we overlaid the local resolution maps onto the corresponding density maps by ChimeraX (Pettersen et al. 2021), and the results are listed in Supplementary Figure S10.

2.8 Supplementary Note S8: Ablation study

2.8.1 Impact of different task frameworks

To further evaluate the performance gain brought by the proposed multi-task framework for particle picking, we designed two ablation models. To validate the benefit of task collaboration, one executes denoising and prompt generation sequentially, where both modules are cascaded without the SFI and multi-task joint training (W/O MTL). Aiming to analyze the impact of the feature-sharing mechanism, the other replaces the selective feature integrator with a simple channel concatenation module (W/O SFI). Supplementary Table S4 presents the precision, recall, and F1-score of the three models on the particle picking task, along with the results of statistical test.

As shown in Supplementary Table S4, CryoPromptSeg outperforms the other two models in precision, recall, and F1-score. Since statistical tests show p-value<0.05, the improvements are statistically significant. Compared to the W/O MTL model, the multi-task framework achieves average increases of approximately 5%, 9%, and 8% in precision, recall, and F1-score, respectively, highlighting the benefits of task collaboration. Additionally, a well-designed feature-sharing strategy can increase model performance: CryoPromptSeg achieves a 9% increase in precision and over 10% in recall and F1-score compared to simple channel concatenation, demonstrating the effectiveness of the selective feature integrator in promoting inter-task collaboration. To intuitively compare the advantages of performance of the multi-task framework, we employ Supplementary Figure S11 to visualize the denoised images and prompt generation results from the W/O MTL, W/O SFI, and CryoPromptSeg models, respectively.

From the denoised images (top row of Figure S11), the output of the W/O MTL model without semantic information exhibits overly smoothed particle regions and non-prominent particle boundaries. Although the W/O SFI model adopts a multi-task learning strategy that makes particles relatively prominent, the absence of an effective feature-sharing mechanism negatively affects the denoising process, leading to noticeable residual background noise. In contrast, the proposed CryoPromptSeg innovatively integrates semantic guidance with the selective feature integrator, which not only preserves clear and sharp particle edges but also significantly reduces background noise, thereby achieving superior denoising performance.

From the prompt generation results (bottom row of Figure S11), the W/O SFI model produces relatively sparse prompt points (approximately 47), with several corresponding mask prompts missing or exhibiting incomplete boundaries. Although the W/O MTL model generates more prompt points (approximately 68), it similarly suffers from missing or incomplete mask prompts. In contrast, CryoPromptSeg generates a greater number of prompt points (approximately 84) with precise localization, and the mask prompts align well with the points, exhibiting complete mask boundaries.

The denoised images and prompt generation results above demonstrate that the proposed multi-task framework effectively increases the collaboration between the denoising and prompt generation tasks. By introducing the selective feature integrator, it enables efficient feature sharing and facilitates beneficial information exchange between tasks, thereby further increasing the overall model performance.

2.8.2 Impact of different fine-tuning techniques

To evaluate if the SAM model fine-tuned with multi-cognitive visual adapter (Mona SAM) addresses the first scientific question, we compared two fine-tuning methods. The first method directly fine-tunes the SAM model by updating only the prompt encoder and mask decoder parameters (FT SAM). The second method (Adapter SAM) uses the adapter module proposed by Wu et al. (Wu et al. 2025) to replace Mona. Also, Adapter SAM fine-tunes the parameters of the adapter, prompt encoder, and mask decoder.

In addition, the experiment uses the performance of the original pretrained SAM model (Pretrained SAM) on cryo-EM images as the baseline. All experiments input the denoised image and prompts to SAM, and segmentation quality is evaluated by Dice score. The results and statistical tests (Supplementary Table S5) show that since Pretrained SAM fails to segment datasets with small particle size and dense distribution (10345, 10093, and 10017), it is unsuitable to directly segment cryo-EM images. Furthermore, FT SAM, Adapter SAM, and Mona SAM outperform Pretrained SAM across all datasets, indicating that fine-tuning enhances SAM's segmentation performance. Additionally, Mona SAM consistently achieves statistically significant greater Dice score than FT SAM and Adapter SAM across all datasets

(see Supplementary Table S5). It performs better on datasets with small particles (10093 and 10017), suggesting that Mona SAM excels at segmenting small targets.

Overall, the Mona fine-tuning technique effectively increases the image encoder's ability to perceive and represent small targets, thereby increasing the model's segmentation performance for complex cryo-EM images.

2.8.3 Impact of different prompt types

To evaluate the impact of different prompt types on particle picking performance, we conducted an ablation study to compare three settings: point-only prompt, mask-only prompt, and combined prompts (point and mask). Each prompt type was fed into SAM to obtain segmentation result. Supplementary Table S6 lists the performance of the three prompt types in terms of Precision, Recall, F1-score, and Dice score. Supplementary Table S7 also presents the paired T-test results for point-only vs. combined and mask-only vs. combined prompts.

The experimental results show that combined prompts (point and mask) achieve the best overall performance. On most datasets, combined prompts obtain the greatest Precision, Recall, F1-score, and Dice score, with smaller standard deviations. Moreover, compared with point-only and mask-only, the improvements of combined prompts across all metrics are statistically significant ($p \leq 0.05$). This indicates that the combined strategy achieves both great accuracy and robustness, effectively integrating the positional information provided by point and mask prompt. In contrast, point-only prompt outperforms mask-only prompt overall, achieving greater Precision and Recall, which suggests stronger localization capability. Mask-only prompt, however, is more sensitive to mask quality and thus exhibits relatively lower stability. Overall, the combined prompt strategy effectively leverages the complementary strengths of both prompt types, significantly increasing the accuracy of SAM in particle segmentation task.

2.9 Supplementary Note S9: Performance under different complexity scenarios

To analyze the performance of CryoPromptSeg under different complexity scenarios, we partitioned the testing dataset along three dimensions: particle size, signal-to-noise ratio (SNR), and particle density. First, in Supplementary Table S8, we report the particle diameter, SNR, and the average number of particles per 1024 × 1024 pixels image for each dataset, where the latter is used to characterize particle density. Based on previous setup, we map all datasets into a three-dimensional feature space defined by size, SNR, and density from the perspective of task complexity and normalize each dimension, after we consider that particle size, SNR, and density jointly affect the difficulty of the particle picking task. Specifically, particle size and SNR are inversely normalized, such that smaller particle size and lower SNR correspond to more challenging scenarios.

In this space, the Euclidean distance from the normalized data point $(D_{norm}, S_{norm}, N_{norm})$ to the origin is used to characterize its overall complexity, and it is computed as follows:

$$\text{Distance} = \sqrt{(D_{norm})^2 + (\,S_{norm})^2 + (N_{norm})^2} \tag{10}$$

Here, D_{norm} denotes the particle diameter after normalization and inversion, S_{norm} denotes the SNR after normalization and inversion, and N_{norm} denotes the density after normalization.

In Supplementary Figure S12, we visualize this three-dimensional feature space, where the size of each sphere represents the distance of the data point from the origin. The longer is the distance, the larger is the sphere. And then, the task is more challenging. Meanwhile, the color of each sphere indicates the F1-score of CryoPromptSeg on the corresponding dataset. Based on sphere size, Figure S12 shows that datasets can be divided into three levels corresponding to low, medium, and high complexity scenarios. We labeled these three levels as 1, 2, and 3 in the figure S12.

In the low-complexity scenario (EMPIAR-10028), the data point is close to the origin due to a large particle size and high SNR, and CryoPromptSeg achieves a high F1-score of 0.819. The medium-complexity level (EMPIAR-10081, 10345, 10532, 10093) corresponds to moderate particle size or SNR conditions. Although performance slightly decreases, Table I in the main text demonstrates that CryoPromptSeg still outperforms other methods. The high-

complexity scenario mainly includes low SNR, small and high-density particles. The corresponding data points are located far away from the origin. Notably, in the low-SNR dataset (EMPIAR-11056), the F1-score reaches 0.748; in the small and high-density dataset (EMPIAR-10017), it is 0.727, both comparable to that of medium-complexity datasets (e.g., EMPIAR-10532, 0.729). This result indicates that CryoPromptSeg can maintain stable performance in typical challenging scenarios involving small particle, low SNR, and high density, without a significant performance degradation.

2.10 Supplementary Note S10: Training and inference time comparison

We compared the training and inference time of CrYOLO, Topaz, CryoTransformer, CryoSegNet, and CryoPromptSeg under the same CPU core and GPU settings by Supplementary Table S9. The training time is defined as the time requested to complete one epoch on the same training dataset, whereas the inference time denotes the average processing time per micrograph. Since our method simultaneously performs image denoising and particle picking during both training and inference, it incurs relatively high computational cost, but achieves superior accuracy (Table Ⅰ of the main text).

3. Supplementary Figures

3.1 Distribution of Recall in different datasets

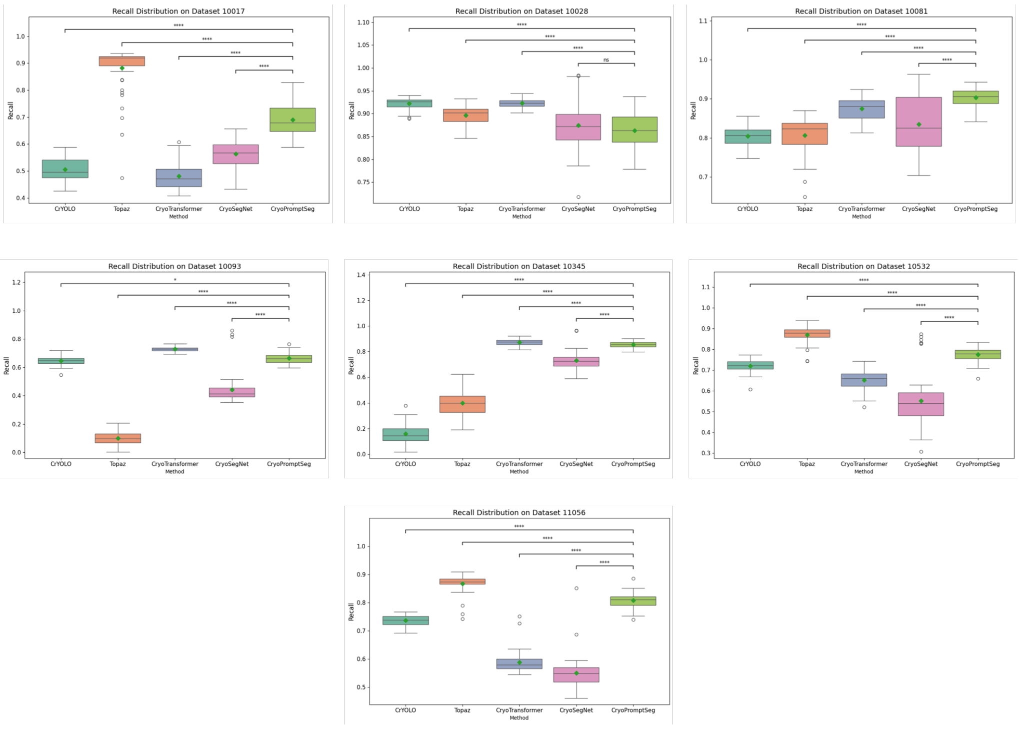

Supplementary Figure S1: Recall distributions of cryo-EM image datasets from seven different proteins (EMPIAR-10017, 10028, 10081, 10093, 10345, 10532, 11056) under different particle picking methods.

Each subplot corresponds to one dataset. The central line indicates the median, green diamonds denote the mean, and circles represent outliers, respectively. Significance annotations above the plots indicate the results of paired T-tests between CryoPromptSeg and other methods. The significance levels are represented as follows: ns$(p > 0.05)$、$*$ $(p \leq 0.05)$、$**$ $(p \leq 0.01)$、$***$ $(p \leq 0.001)$、$****$ $(p \leq 0.0001)$.

3.2 Distribution of F1-score in different datasets

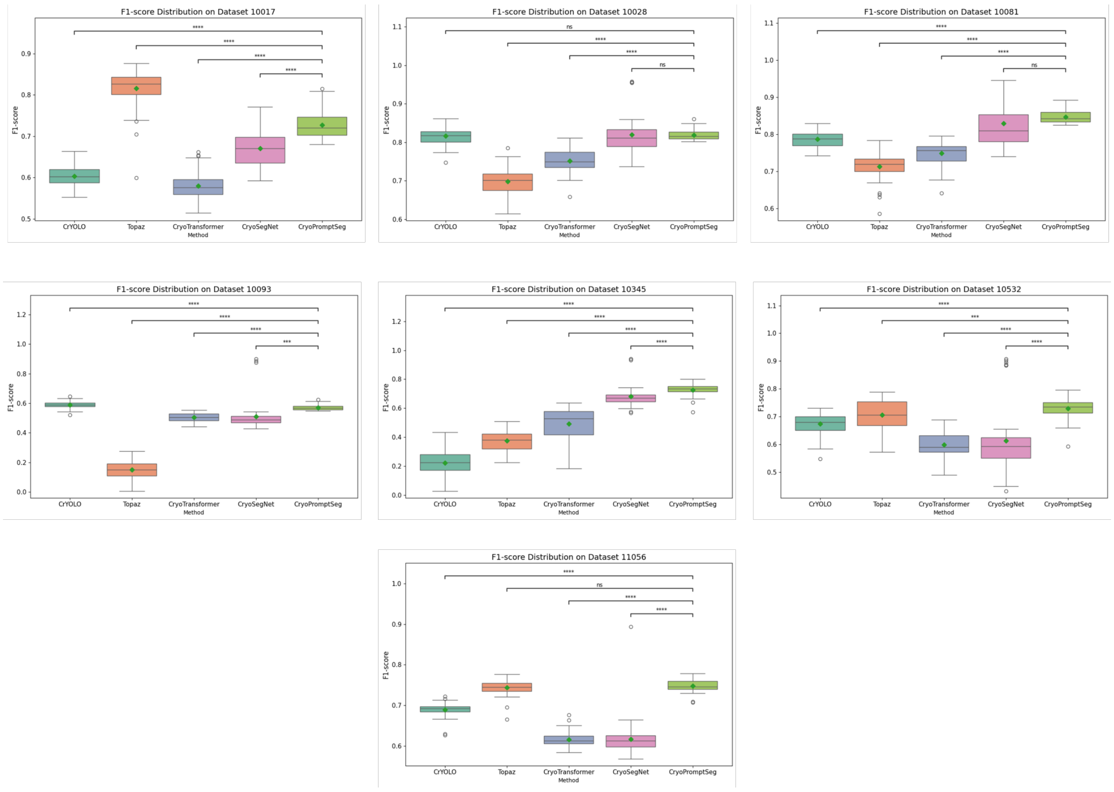

Supplementary Figure S2: F1-score distributions of cryo-EM image datasets from seven different proteins (EMPIAR-10017, 10028, 10081, 10093, 10345, 10532, 11056) under different particle picking methods.

Each subplot corresponds to one dataset. The central line indicates the median, green diamonds denote the mean, and circles represent outliers, respectively. Significance annotations above the plots indicate the results of paired T-tests between CryoPromptSeg and other methods. The significance levels are represented as follows: ns($p > 0.05$)、$*$ ($p \le 0.05$)、$**$ ($p \le 0.01$)、$***$ ($p \le 0.001$)、$****$ ($p \le 0.0001$).

3.3 Distribution of Dice score in different datasets

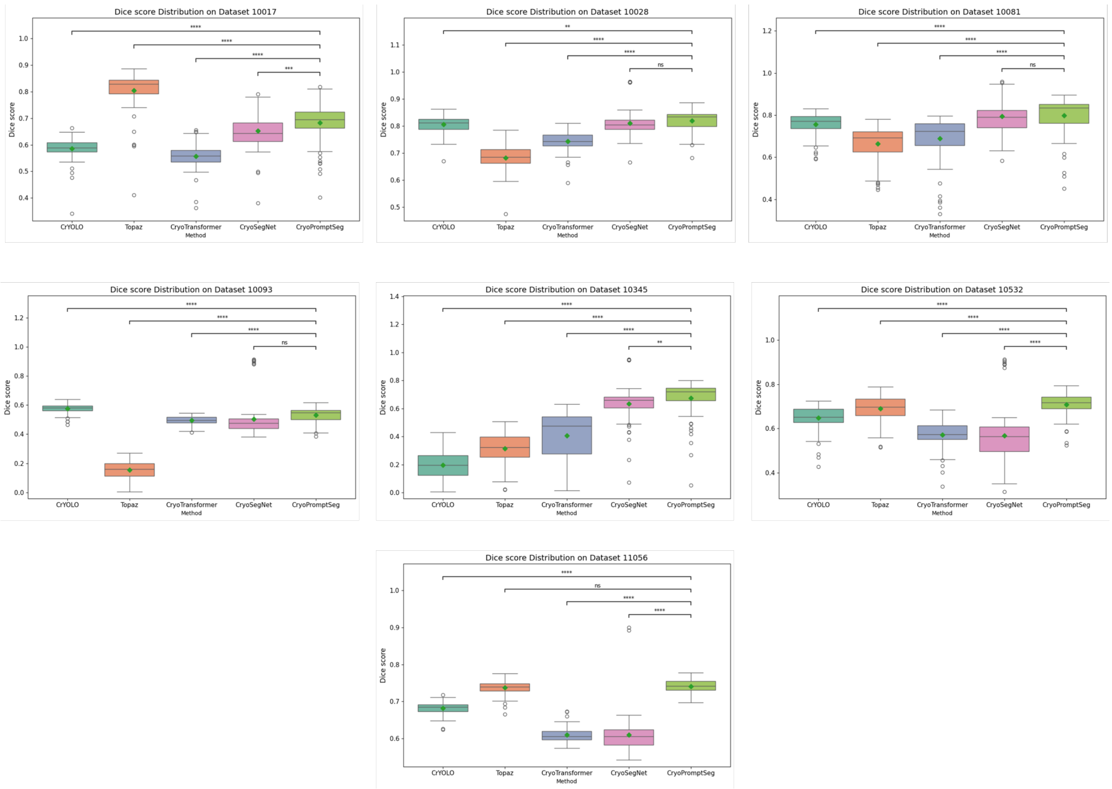

Supplementary Figure S3: Dice score distributions of cryo-EM image datasets from seven different proteins (EMPIAR-10017, 10028, 10081, 10093, 10345, 10532, 11056) under different particle picking methods.

Each subplot corresponds to one dataset. The central line indicates the median, green diamonds denote the mean, and circles represent outliers, respectively. Significance annotations above the plots indicate the results of paired T-tests between CryoPromptSeg and other methods. The significance levels are represented as follows: ns($p > 0.05$)、$*$ ($p \leq 0.05$)、$**$ ($p \leq 0.01$)、$***$ ($p \leq 0.001$)、$****$ ($p \leq 0.0001$).

3.4 Distribution of SNR in different datasets

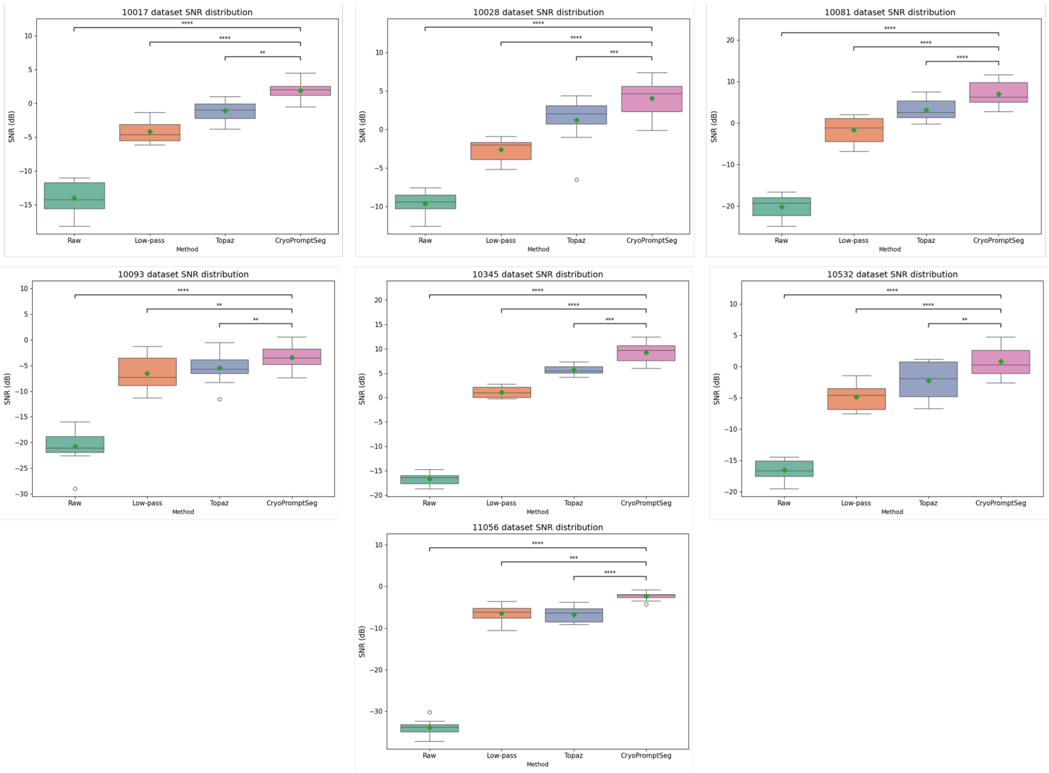

Supplementary Figure S4: Signal-to-noise ratio (SNR) distributions of cryo-EM image datasets from seven different proteins (EMPIAR-10017, 10028, 10081, 10093, 10345, 10532, 11056) under different denoising methods (including raw images).

Each subplot corresponds to one dataset, and the box plot illustrates the distribution of SNR values obtained with different denoising methods. The central line indicates the median, green diamonds denote the mean, and circles represent outliers, respectively. Significance annotations above the plots indicate the results of paired *T*-tests between CryoPromptSeg and other methods. The significance levels are represented as follows: ns($p > 0.05$)、$*$ ($p \leq 0.05$)、$**$ ($p \leq 0.01$)、$***$ ($p \leq 0.001$)、$****$ ($p \leq 0.0001$).

3.5 Examples of particle and background regions

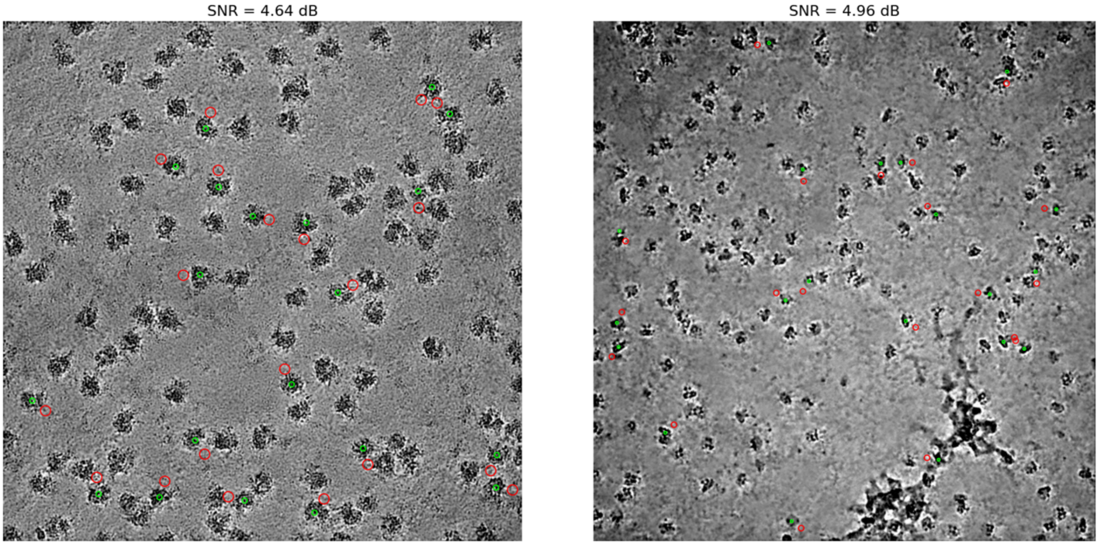

10028 **10081**

Supplementary Figure S5: Examples of particle and background regions used for SNR calculation in the denoising evaluation.

The figure shows cryo-EM images from two datasets (protein 10028 and 10081) after being denoised by CryoPromptSeg. The particle region is indicated by a green circle and the background region is indicated by a red circle, along with the resulting SNR value. Each particle region corresponds to a background region, and the background is chosen as close as possible to the corresponding particle. In addition, since the sizes of different proteins are various each other, the diameters of the selected circles differ accordingly.

3.6 Selected 2D classes of protein particles

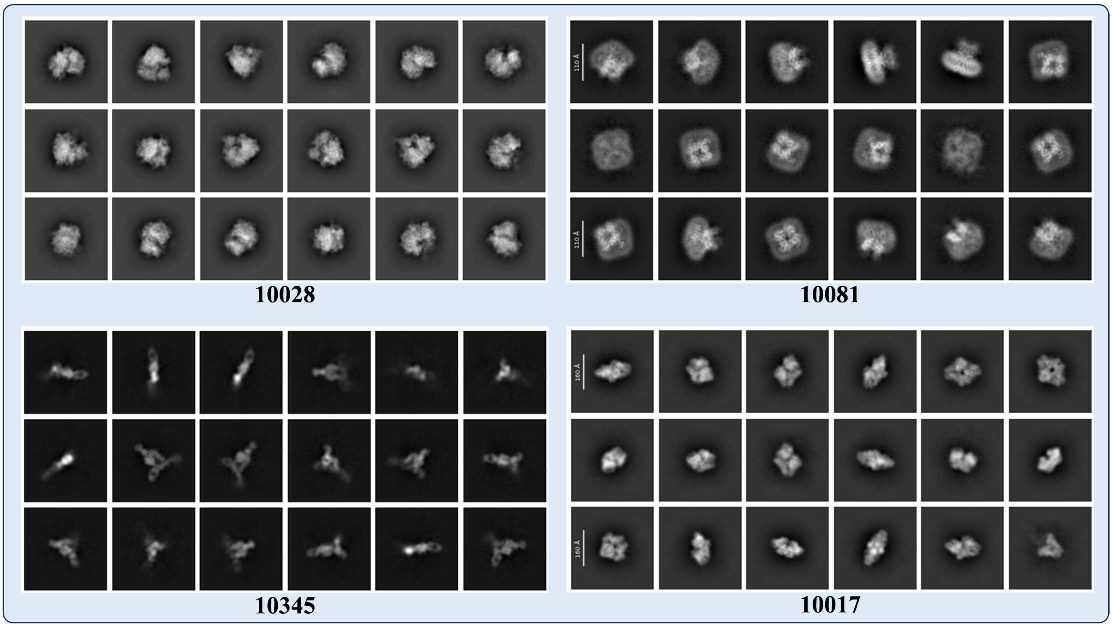

Supplementary Figure S6: 2D classes from particles selected by CryoPromptSeg for the EMPIAR-10028, 10081, 10345, and 10532 datasets.

These classes indicate that CryoPromptSeg can select particles with multiple orientations, encompassing diverse views of the proteins, which is crucial to obtain high-resolution density maps.

3.7 Comparison of 3D density maps

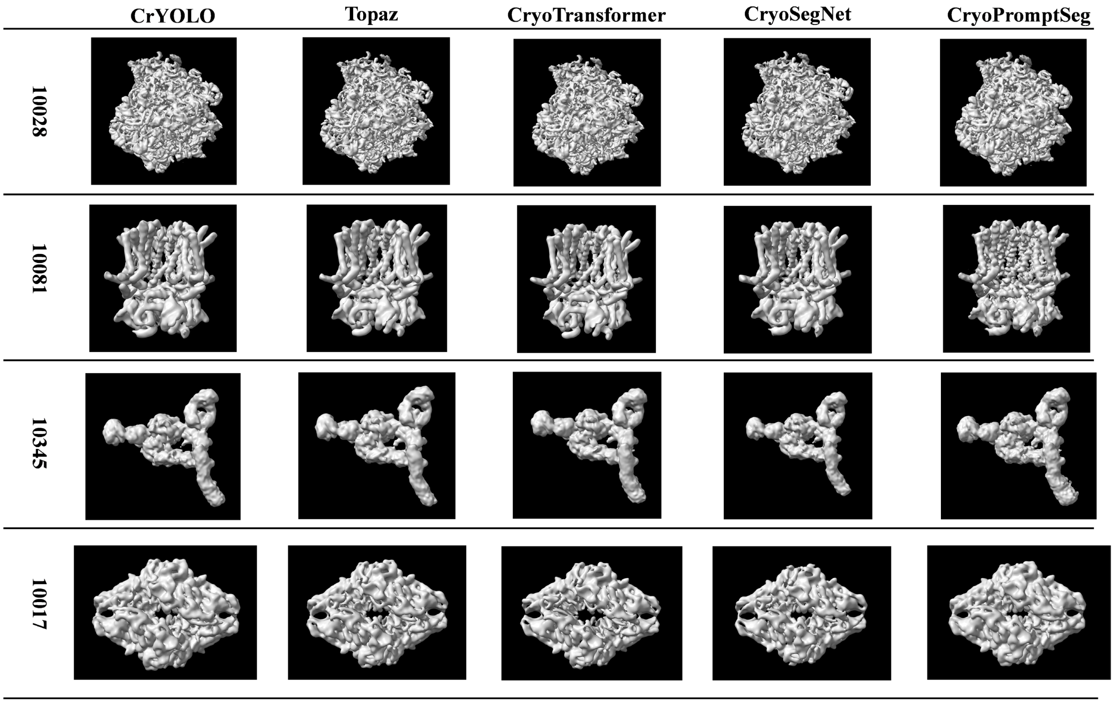

Supplementary Figure S7: Comparison of 3D density maps reconstructed from particles picked by CrYOLO, Topaz, CryoTransformer, CryoSegNet, and CryoPromptSeg.

3.8 Additional reconstruction results

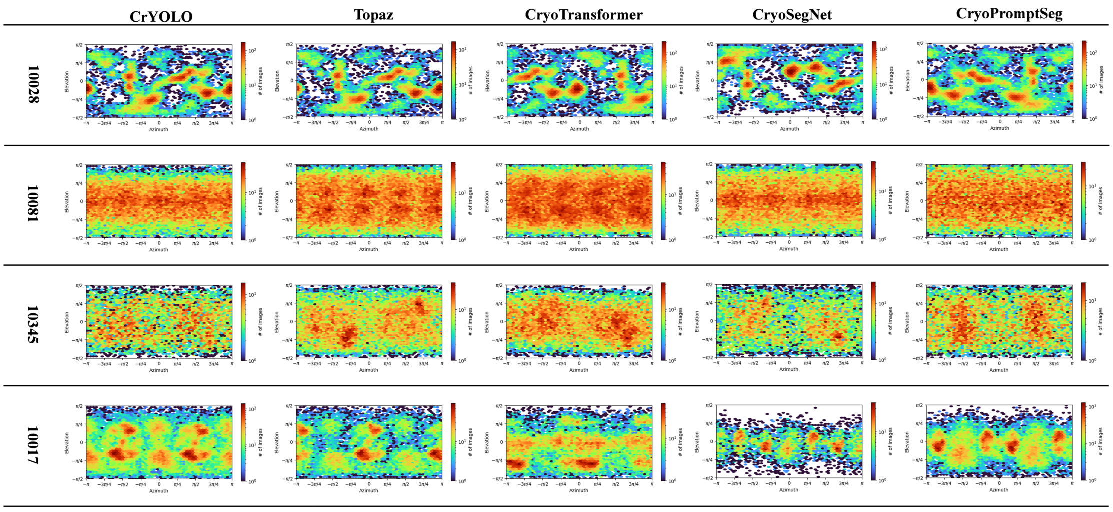

Supplementary Figure S8: Viewing direction plots of four proteins (EMPIAR-10028, 10081, 10345, and 10532). The viewing direction plot with elevation and azimuth as coordinates; higher red intensity indicates a greater number of particles in that particular elevation versus azimuth direction.

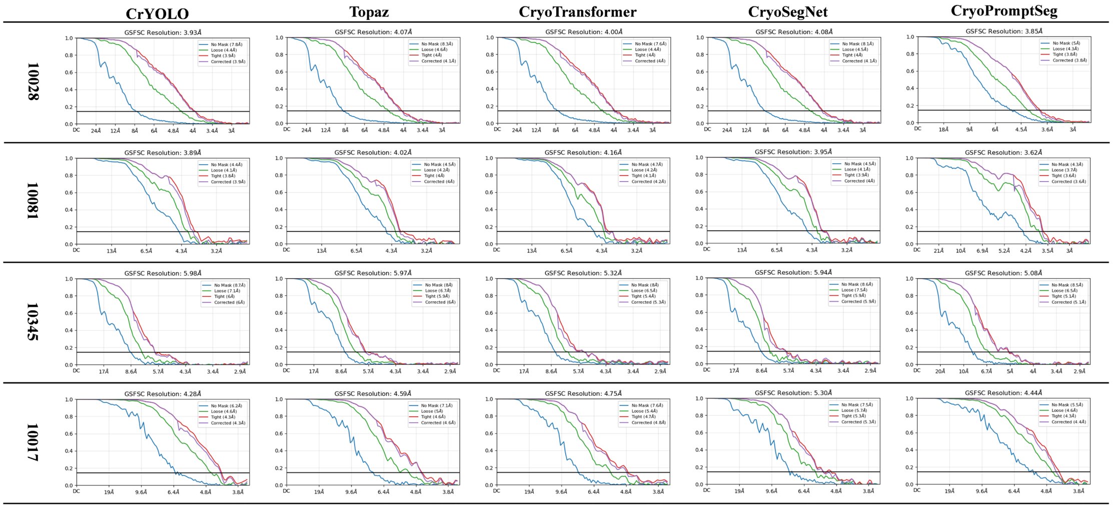

Supplementary Figure S9: 3D resolution CSFSC curves of four proteins (EMPIAR-10028, 10081, 10345, and 10532). The CSFSC curve characterizes the resolution of the 3D density map reconstructed from the selected particles.

3.9 Comparison of local resolution estimation

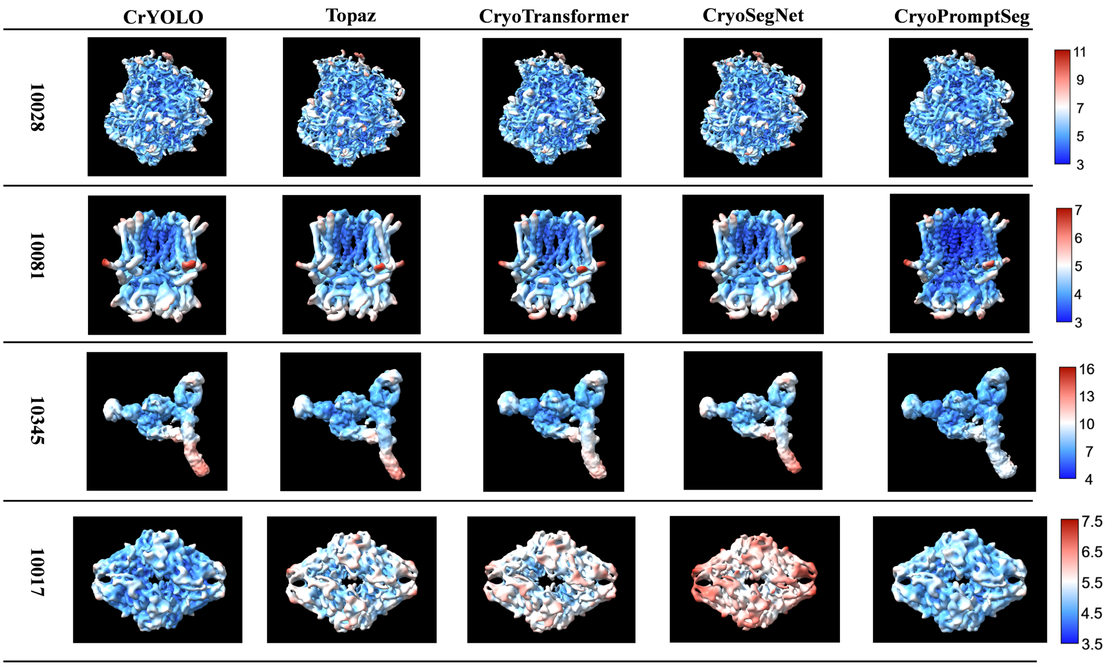

Supplementary Figure S10: Comparison results for local resolution estimation of density maps of particles picked by CrYOLO, Topaz, CryoTransformer, CryoSegNet and CryoPromptSeg. The color bar shown on the right of each row (in Å) indicates the resolution scale, where blue represents regions of higher resolution and red represents regions of lower resolution.

3.10 Visualization results of denoising and prompt generation

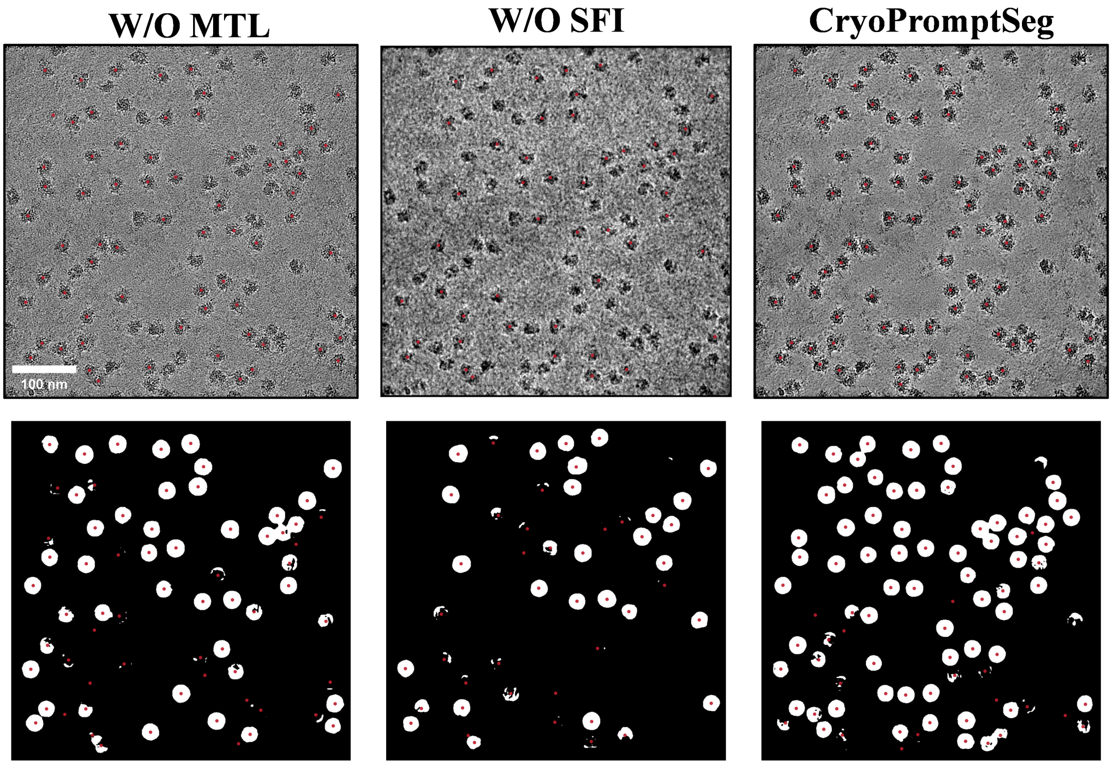

Supplementary Figure S11: Visualization results of the denoising and prompt generation tasks. Top row: denoised images overlaid with prompt points, where prompt points are marked in red; Bottom row: mask prompts overlaid with prompt points, where prompt points marked in red.

3.11 Visualization of the three-dimensional feature space

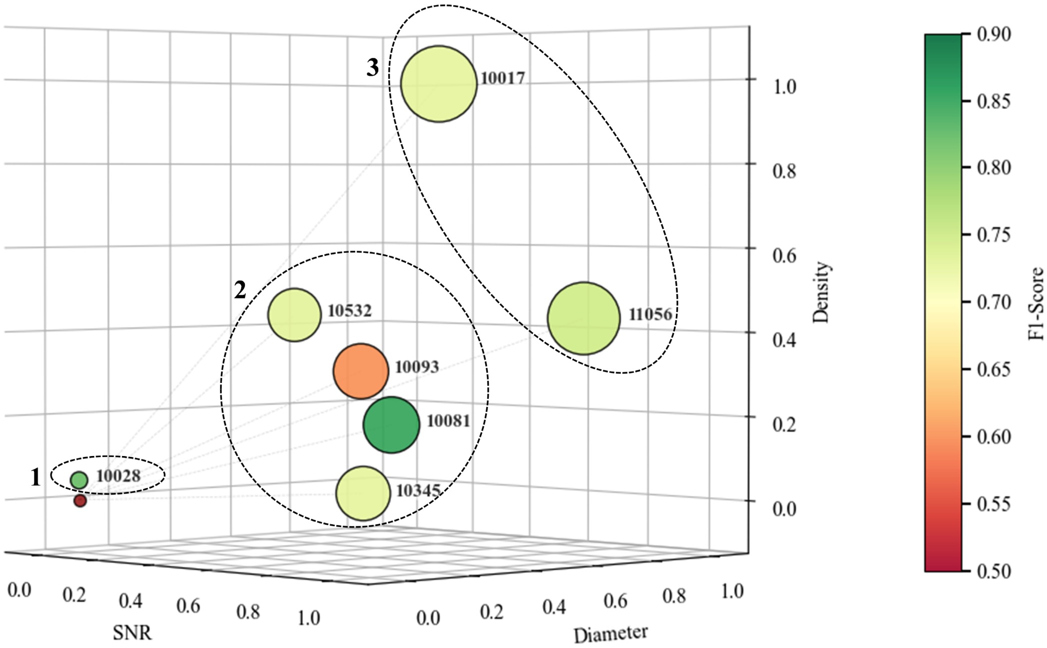

Supplementary Figure S12: Visualization of the three-dimensional feature space. The size of each sphere represents the distance of the data point from the origin—the longer the distance, the larger the sphere and the greater the task complexity. The color of each sphere indicates the F1-score of CryoPromptSeg on the corresponding dataset. Based on sphere size, the datasets can be divided into three levels: 1, 2, and 3, corresponding to low, medium, and high complexity scenarios, respectively.

References

Asami J, Kimura K T, Fujita-Fujiharu Y *et al.* Structure of the bile acid transporter and HBV receptor NTCP. *Nature* 2022;606:1021-1026.

Bepler T, Morin A, Rapp M *et al.* Positive-unlabeled convolutional neural networks for particle picking in cryo-electron micrographs. *Nat. Methods* 2019;16:1153-1160.

Burendei B, Shinozaki R, Watanabe M *et al.* Cryo-EM structures of undocked innexin-6 hemichannels in phospholipids. *Sci. Adv.* 2020;6:eaax3157.

Campbell M G, Cormier A, Ito S *et al.* Cryo-EM reveals integrin-mediated TGF-β activation without release from latent TGF-β. *Cell* 2020;180:490-501. e416.

Cao C, Kang H J, Singh I *et al.* Structure, function and pharmacology of human itch GPCRs. *Nature* 2021;600:170-175.

Demura K, Kusakizako T, Shihoya W *et al.* Cryo-EM structures of calcium homeostasis modulator channels in diverse oligomeric assemblies. *Sci. Adv.* 2020;6:eaba8105.

Dhakal A, Gyawali R, Wang L *et al.* CryoTransformer: a transformer model for picking protein particles from cryo-EM micrographs. *Bioinformatics* 2024;40.

Fischer N, Neumann P, Bock L V *et al.* The pathway to GTPase activation of elongation factor SelB on the ribosome. *Nature* 2016;540:80-85.

Gao Y, Cao E, Julius D *et al.* TRPV1 structures in nanodiscs reveal mechanisms of ligand and lipid action. *Nature* 2016;534:347-351.

Gyawali R, Dhakal A, Wang L *et al.* CryoSegNet: accurate cryo-EM protein particle picking by integrating the foundational AI image segmentation model and attention-gated U-Net. *Brief. Bioinform.* 2024;25.

He L, Ren X, Gao Q *et al.* The connected-component labeling problem: A review of state-of-the-art algorithms. *Pattern Recognit.* 2017;70:25-43.

Jin P, Bulkley D, Guo Y *et al.* Electron cryo-microscopy structure of the mechanotransduction channel NOMPC. *Nature* 2017;547:118-122.

Kirillov A, Mintun E, Ravi N *et al.* Segment anything. In: Proceedings of the IEEE/CVF international conference on computer vision, 2023, 4015-4026.

Koning R I, Gomez-Blanco J, Akopjana I *et al.* Asymmetric cryo-EM reconstruction of phage MS2 reveals genome structure in situ. *Nat. Commun.* 2016;7:12524.

Kuzuya M, Hirano H, Hayashida K *et al.* Structures of human pannexin-1 in nanodiscs reveal gating mediated by dynamic movement of the N terminus and phospholipids. *Sci. Signal.* 2022;15:eabg6941.

Lee C-H and MacKinnon R J C. Structures of the human HCN1 hyperpolarization-activated channel. *Cell* 2017;168:111-120. e111.

Li J, Han L, Vallese F *et al.* Cryo-EM structures of Escherichia coli cytochrome bo 3 reveal bound phospholipids and ubiquinone-8 in a dynamic substrate binding site. *Proc. Natl. Acad. Sci. U. S. A.* 2021;118:e2106750118.

Liao M, Cao E, Julius D *et al.* Structure of the TRPV1 ion channel determined by electron cryo-microscopy. *Nature* 2013;504:107-112.

Liu Y, Cao C, Huang X-P *et al.* Ligand recognition and allosteric modulation of the human MRGPRX1 receptor. *Nat. Chem. Biol.* 2023;19:416-422.

Mashtalir N, Suzuki H, Farrell D P *et al.* A structural model of the endogenous human BAF complex

informs disease mechanisms. *Cell* 2020;183:802-817. e824.

Newing T P, Oakley A J, Miller M *et al.* Molecular basis for RNA polymerase-dependent transcription complex recycling by the helicase-like motor protein HelD. *Nat. Commun.* 2020;11:6420.

Nicholson D, Edwards T A, O'Neill A J *et al.* Structure of the 70S ribosome from the human pathogen Acinetobacter baumannii in complex with clinically relevant antibiotics. *Structure* 2020;28:1087-1100. e1083.

Oldham M L, Grigorieff N and Chen J J E. Structure of the transporter associated with antigen processing trapped by herpes simplex virus. *Elife* 2016;5:e21829.

Pettersen E F, Goddard T D, Huang C C *et al.* UCSF ChimeraX: Structure visualization for researchers, educators, and developers. *Protein Sci* 2021;30:70-82.

Punjani A, Rubinstein J L, Fleet D J *et al.* CryoSPARC: algorithms for rapid unsupervised cryo-EM structure determination. *Nat. Methods* 2017;14:290-296.

Scheres S H J J o s b. Semi-automated selection of cryo-EM particles in RELION-1.3. *J. Struct. Biol.* 2015;189:114-122.

Tan Y Z, Baldwin P R, Davis J H *et al.* Addressing preferred specimen orientation in single-particle cryo-EM through tilting. *Nat. Methods* 2017;14:793-796.

Tan Y Z and Rubinstein J L J B C. Through-grid wicking enables high-speed cryoEM specimen preparation. *Acta Crystallogr. Sect. D-Biol. Crystallogr.* 2020;76:1092-1103.

Wagner T, Merino F, Stabrin M *et al.* SPHIRE-crYOLO is a fast and accurate fully automated particle picker for cryo-EM. *Commun. Biol.* 2019;2:218.

Wong W, Bai X-c, Brown A *et al.* Cryo-EM structure of the Plasmodium falciparum 80S ribosome bound to the anti-protozoan drug emetine. *elife* 2014;3:e03080.

Wu J, Wang Z, Hong M *et al.* Medical sam adapter: Adapting segment anything model for medical image segmentation. *Med. Image Anal.* 2025;102:103547.
